# Supplementary material for: RAINBOW: Haplotype-based genome-wide association study using a novel SNP-set method
Source: PLoS Comput Biol. 2020 Feb 14;16(2):e1007663. doi: 10.1371/journal.pcbi.1007663 (PMC7046296; doi:10.1371/journal.pcbi.1007663)
Supplement: S1 Appendix — A faster computational method for the linear kernel and effective testing method for dominance and epistatic effects are mainly described. (PDF) [file pcbi.1007663.s001.pdf]

Notations

In this section, we define the settings of the following methods, and introduce the frequently used identities.

- $\mathbf{I}_J$ denotes a $J \times J$ identity matrix.

- $\mathbf{1}_{I \times J}$ denotes a $I \times J$ matrix whose elements are all 1.

- MVN $(\boldsymbol{\mu}, \boldsymbol{\Sigma})$ denotes a multivariate normal distribution with a mean $\boldsymbol{\mu}$ and a variance-covariance matrix $\boldsymbol{\Sigma}$.

- For any $a \times b$ matrix $\mathbf{A}$, the $b \times a$ matrix $\mathbf{A}^\dagger$ denotes the Moore-Penrose pseudo-inverse of $\mathbf{A}$.

- For any $a \times b$ matrix $\mathbf{A}$ and $a \times b$ matrix $\mathbf{B}$, the $a \times b$ matrix $\mathbf{C} = \mathbf{A} \circ \mathbf{B}$ denotes the Hadamard product between $\mathbf{A}$ and $\mathbf{B}$.

- For any $a \times a$ square matrix $\mathbf{A}$, the expression $|\mathbf{A}|_+$ denotes the pseudo-determinant of $\mathbf{A}$. If $\mathbf{A}$ is positive semi-difinite, $|\mathbf{A}|_+$ will be computed as the product of non-zero eigen values of $\mathbf{A}$.

- $\mathbf{y}$ denotes a $n \times 1$ vector of phenotypic values.

- $\mathbf{X}$ denotes a $n \times p$ covariate matrix with full column rank p where $n \geq p$.

- $\boldsymbol{\beta}$ denotes a $p \times 1$ vector of fixed effects for covariates.

- $\mathbf{Z}_\mathrm{c}$ denotes a $n \times m_\mathrm{c}$ design matrix corresponding to random effects $\mathbf{u}_\mathrm{c}$ for family relatedness.

- $\mathbf{u}_\mathrm{c}$ denotes a $m_\mathrm{c} \times 1$ vector of random effects for family relatedness. We assume $\mathbf{u}_\mathrm{c} \sim \mathrm{MVN}(\mathbf{0}, \mathbf{K}_\mathrm{c}\sigma^2_\mathrm{c})$ where $\mathbf{K}_\mathrm{c}$ is the additive genetic relationship matrix estimated from marker genotype $\tilde{\mathbf{W}}_\mathrm{c}$ and σ^2_c is the additive genetic variance.

- $\mathbf{Z}_{\mathrm{r}_i}$ denotes a $n \times m_{\mathrm{r}_i}$ design matrix corresponding to random effects $\mathbf{u}_{\mathrm{r}_i}$ for each SNP-set.

- $\mathbf{u}_{\mathrm{r}_i}$ denotes a $m_{\mathrm{r}_i} \times 1$ vector of random effects for each SNP-set. We assume $\mathbf{u}_{\mathrm{r}_i} \sim \mathrm{MVN}(\mathbf{0}, \mathbf{K}_{\mathrm{r}_i}\sigma^2_{\mathrm{r}_i})$ where $\mathbf{K}_{\mathrm{r}_i}$ is the Gram matrix computed from marker genotype in SNP-set of interest $\tilde{\mathbf{W}}_{\mathrm{r}_i}$ and $\sigma^2_{\mathrm{r}_i}$ is the genetic variance of each SNP-set.

- $\boldsymbol{\epsilon}$ denotes a $n \times 1$ vector of residuals. We assume $\boldsymbol{\epsilon} \sim \mathrm{MVN}(\mathbf{0}, \mathbf{I}_n \sigma_{\mathrm{e}}^2)$ where σ_{e}^2 is the residual variance.

- $\mathbf{V}$ is a $n \times n$ phenotypic variance-covariance matrix. In this paper, $\mathbf{V} = \mathbf{Z}_{\mathrm{c}}\mathbf{K}_{\mathrm{c}}\mathbf{Z}_{\mathrm{c}}^{\mathrm{T}}\sigma_{\mathrm{c}}^2 + \mathbf{Z}_{\mathrm{r}_i}\mathbf{K}_{\mathrm{r}_i}\mathbf{Z}_{\mathrm{r}_i}^{\mathrm{T}}\sigma_{\mathrm{r}_i}^2 + \mathbf{I}_n\sigma_{\mathrm{e}}^2$. Under the null hypothesis $\sigma_{\mathrm{r}_i}^2 = 0$ since $\sigma_{\mathrm{r}_i}^2$ is the parameter to be tested. $\mathbf{V}$ is assumed to be a full rank (n) matrix.

- $\mathbf{H}$ is a $n \times n$ matrix which satisfies $\mathbf{H} = \mathbf{V}/\sigma_{\mathrm{c}}^2 = \mathbf{Z}_{\mathrm{c}}\mathbf{K}_{\mathrm{c}}\mathbf{Z}_{\mathrm{c}}^{\mathrm{T}} + \mathbf{Z}_{\mathrm{r}_i}\mathbf{K}_{\mathrm{r}_i}\mathbf{Z}_{\mathrm{r}_i}^{\mathrm{T}}\gamma_{\mathrm{r}_i} + \mathbf{I}_n\delta_{\mathrm{e}}$ where $\gamma_{\mathrm{r}_i} = \sigma_{\mathrm{r}_i}^2/\sigma_{\mathrm{c}}^2$ and $\delta_{\mathrm{e}} = \sigma_{\mathrm{e}}^2/\sigma_{\mathrm{c}}^2$ to be estimated.

- $\mathbf{S} = \mathbf{I}_n - \mathbf{X}\left(\mathbf{X}^{\mathrm{T}}\mathbf{X}\right)^{-1}\mathbf{X}^{\mathrm{T}}$ is a $n \times n$ symmetric covariance orthogonal projection matrix with rank $n - p$.

- $\mathbf{P} = \mathbf{I}_n - \mathbf{X}\left(\mathbf{X}^{\mathrm{T}}\mathbf{V}^{-1}\mathbf{X}\right)^{-1}\mathbf{X}^{\mathrm{T}}\mathbf{V}^{-1} = \mathbf{I}_n - \mathbf{X}\left(\mathbf{X}^{\mathrm{T}}\mathbf{H}^{-1}\mathbf{X}\right)^{-1}\mathbf{X}^{\mathrm{T}}\mathbf{H}^{-1}$ is a $n \times n$ matrix with rank $n - p$.

- $\mathbf{w} = \mathbf{P}\mathbf{y}$ is a $n \times 1$ vector used for the REML estimation. We also define $\mathbf{w}_{\mathrm{L}}$, $\mathbf{w}_{\mathrm{R}}$, $\mathbf{P}_{\mathrm{L}}$ and $\mathbf{P}_{\mathrm{R}}$ by

  $$\mathbf{w} = \begin{bmatrix}\mathbf{w}_{\mathrm{L}} \\ \mathbf{w}_{\mathrm{R}}\end{bmatrix} = \begin{bmatrix}\mathbf{P}_{\mathrm{L}} \\ \mathbf{P}_{\mathrm{R}}\end{bmatrix}\mathbf{y} = \mathbf{P}\mathbf{y},$$

  where $\mathbf{w}_{\mathrm{L}}$ (or $\mathbf{w}_{\mathrm{R}}$) stands for a linearly independent (or redundant) part of $\mathbf{w}$, and $\mathbf{P}_{\mathrm{L}}$ (or $\mathbf{P}_{\mathrm{R}}$) denotes the corresponding partition of $\mathbf{P}$. Here, $\mathbf{P}_{\mathrm{L}}$ should satisfy $\mathbf{P}_{\mathrm{L}}^{\mathrm{T}}\mathbf{P}_{\mathrm{L}} = \mathbf{S}$ and $\mathbf{P}_{\mathrm{L}}\mathbf{P}_{\mathrm{L}}^{\mathrm{T}} = \mathbf{I}_{n-p}$ [1, 2, 3]. We also define the eigen decomposition of $\mathbf{SHS}$ as

  $$\mathbf{SHS} = \mathbf{U}_{\mathrm{L}}\boldsymbol{\Lambda}_L\mathbf{U}_{\mathrm{L}}^{\mathrm{T}},$$

  where $\boldsymbol{\Lambda}_L$ is a $n - p \times n - p$ diagnonal matrix whose elements are non-zero eigen values of $\mathbf{SHS}$ in the decreasing order, and $\mathbf{U}_{\mathrm{L}}$ is a $n \times n - p$ eigen vector matrix whose each eigen vector corresponds to each eigen value. Here, $\mathbf{U}_{\mathrm{L}}$ is the part of the unitary matrix with first $n - p$ columns of eigen vectors, and it satisfies $\mathbf{U}_{\mathrm{L}}\mathbf{U}_{\mathrm{L}}^{\mathrm{T}} = \mathbf{S}$ and $\mathbf{U}_{\mathrm{L}}^{\mathrm{T}}\mathbf{U}_{\mathrm{L}} = \mathbf{I}_{n-p}$ (Proposition 7). Therefore, we can say $\mathbf{P}_{\mathrm{L}} = \mathbf{U}_{\mathrm{L}}^{\mathrm{T}}$.

- $\mathbf{Q} = \mathbf{H}^{-1} - \mathbf{H}^{-1}\mathbf{X}\left(\mathbf{X}^{\mathrm{T}}\mathbf{H}^{-1}\mathbf{X}\right)^{-1}\mathbf{X}^{\mathrm{T}} = \mathbf{H}^{-1}\mathbf{P} = \mathbf{P}^{\mathrm{T}}\mathbf{H}^{-1}$ is a $n \times n$ symmetry matrix with rank $n - p$.

- $\mathbf{W}_{\mathrm{r}_i}$ is a $m_{\mathrm{r}_i} \times M_i$ marker genotype matrix belonging to the i-th SNP-set. Here, M_i is the number of SNPs in the i-th SNP-set.

Multi-kernel linear mixed model

In this study, the alternative model can be written as the multi-kernel mixed model.

$$\mathbf{y} = \mathbf{X}\boldsymbol{\beta} + \mathbf{Z}_{\mathrm{c}}\mathbf{u}_{\mathrm{c}} + \mathbf{Z}_{\mathrm{r}_i}\mathbf{u}_{\mathrm{r}_i} + \boldsymbol{\epsilon} \tag{1}$$

On the other hand, the null model can be written as

$$\mathbf{y} = \mathbf{X}\boldsymbol{\beta} + \mathbf{Z}_{\mathrm{c}}\mathbf{u}_{\mathrm{c}} + \boldsymbol{\epsilon} \tag{2}$$

Therefore, we somehow test the null hypothesis $H_0 : \sigma^2_{\mathrm{r}_i} = 0$ for evaluating the significance of the effects of SNP-set of interest.

Restricted maximum likelihood (REML)

For the multi-kernel linear mixed model described above, the restricted log likelihood of $\mathbf{y}$ can be regarded as the log likelihood of $\mathbf{w}_{\mathrm{L}}$, and it can be expressed as a quite simple format by only using $\mathbf{Q}$.

$$\begin{aligned} l_{\mathrm{R}}(\mathbf{y}; \sigma_{\mathrm{c}}, \gamma_{\mathrm{r}_i}, \delta_{\mathrm{e}}) &= -\frac{n-p}{2}\log(2\pi) - \frac{1}{2}\log\left|\mathbf{P}_{\mathrm{L}}\mathbf{V}\mathbf{P}_{\mathrm{L}}^{\mathrm{T}}\right| - \frac{1}{2}\mathbf{y}\mathbf{P}_{\mathrm{L}}^{\mathrm{T}}\left(\mathbf{P}_{\mathrm{L}}\mathbf{V}\mathbf{P}_{\mathrm{L}}^{\mathrm{T}}\right)^{-1}\mathbf{P}_{\mathrm{L}}\mathbf{y} \\ &= -\frac{n-p}{2}\log(2\pi) + \frac{1}{2}\log\left|\mathbf{P}^{\mathrm{T}}\mathbf{V}^{-1}\mathbf{P}\right|_{+} - \frac{1}{2}\mathbf{y}^{\mathrm{T}}\mathbf{V}^{-1}\mathbf{P}\mathbf{y} \\ &= -\frac{n-p}{2}\log(2\pi\sigma_{\mathrm{c}}^2) + \frac{1}{2}\log|\mathbf{Q}|_{+} - \frac{1}{2\sigma_{\mathrm{c}}^2}\mathbf{y}^{\mathrm{T}}\mathbf{Q}\mathbf{y} \end{aligned} \tag{3}$$

by using Proposition 8 and 9.
In addition, by using Proposition 10, this restricted log likelihood is same as the well known format [4, 5, 6] as follows.

$$\begin{aligned} l_{\mathrm{R}}(\mathbf{y}; \sigma_{\mathrm{c}}, \gamma_{\mathrm{r}_i}, \delta_{\mathrm{e}}) &= -\frac{n-p}{2}\log(2\pi\sigma_{\mathrm{c}}^2) + \frac{1}{2}\log|\mathbf{Q}|_{+} - \frac{1}{2\sigma_{\mathrm{c}}^2}\mathbf{y}^{\mathrm{T}}\mathbf{Q}\mathbf{y} \\ &= -\frac{n-p}{2}\log(2\pi\sigma_{\mathrm{c}}^2) - \frac{1}{2}\log|\mathbf{H}| + \frac{1}{2}\log\left|\mathbf{X}^{\mathrm{T}}\mathbf{X}\right| \\ &\quad - \frac{1}{2}\log\left|\mathbf{X}^{\mathrm{T}}\mathbf{H}^{-1}\mathbf{X}\right| - \frac{1}{2\sigma_{\mathrm{c}}^2}\mathbf{y}^{\mathrm{T}}\mathbf{Q}\mathbf{y} \end{aligned} \tag{4}$$

Plugging $\hat{\sigma}_{\mathrm{c}}^2 = \mathbf{y}^{\mathrm{T}}\mathbf{Q}\mathbf{y}/(n-p)$ into Eq 3, we get

$$l_{\mathrm{R}}(\mathbf{y}; \hat{\sigma}_{\mathrm{c}}, \gamma_{\mathrm{r}_i}, \delta_{\mathrm{e}}) = -\frac{n-p}{2}\left\{\log\left(\frac{2\pi e}{n-p}\right) + \log\left(\mathbf{y}^{\mathrm{T}}\mathbf{Q}\mathbf{y}\right)\right\} + \frac{1}{2}\log|\mathbf{Q}|_{+} \tag{5}$$

Efficient likelihood ratio test used in RAINBOW

In this section, we describe how to implement computationally efficient algorithm for the likelihood ratio (LR) test [7, 8] for the two kernel linear mixed model.

In this study, we assume the Gram matrix for each SNP-set $\mathbf{Z}_{\mathrm{r}_i}\mathbf{K}_{\mathrm{r}_i}\mathbf{Z}_{\mathrm{r}_i}^{\mathrm{T}}$ is low rank, so *rank*$(\mathbf{Z}_{\mathrm{r}_i}\mathbf{K}_{\mathrm{r}_i}\mathbf{Z}_{\mathrm{r}_i}^{\mathrm{T}}) \ll n$. Then, $\mathbf{H}$ can be written as

$$\begin{aligned} \mathbf{H} &= \mathbf{Z}_{\mathrm{c}}\mathbf{K}_{\mathrm{c}}\mathbf{Z}_{\mathrm{c}}^{\mathrm{T}} + \mathbf{Z}_{\mathrm{r}_i}\mathbf{K}_{\mathrm{r}_i}\mathbf{Z}_{\mathrm{r}_i}^{\mathrm{T}}\gamma_{\mathrm{r}_i} + \mathbf{I}_n\delta_{\mathrm{e}} \\ &= \mathbf{Z}_{\mathrm{c}}\mathbf{K}_{\mathrm{c}}\mathbf{Z}_{\mathrm{c}}^{\mathrm{T}} + \tilde{\mathbf{W}}_{\mathrm{r}_i}\tilde{\boldsymbol{\Gamma}}_{\mathrm{r}_i}\tilde{\mathbf{W}}_{\mathrm{r}_i}^{\mathrm{T}} + \mathbf{I}_n\delta_{\mathrm{e}}, \end{aligned} \tag{6}$$

where $\tilde{\mathbf{W}}_{\mathrm{r}_i}$ is a $n \times k$ matrix and $\tilde{\boldsymbol{\Gamma}}_{\mathrm{r}_i}$ is a $k \times k$ square matrix. Here, k is the rank of $\mathbf{Z}_{\mathrm{r}_i}\mathbf{K}_{\mathrm{r}_i}\mathbf{Z}_{\mathrm{r}_i}^{\mathrm{T}}$, so $k \ll n$. Concrete examples of $\tilde{\mathbf{W}}_{\mathrm{r}_i}$ and $\tilde{\boldsymbol{\Gamma}}_{\mathrm{r}_i}$ will be described later.

Low rank update of $\mathbf{Q}$

One of the drawbacks of the LR test is a large amount of computation because the LR test requires the maximization of restricted likelihood for each SNP-set. To reduce the computational complexity, [9, 10] proposed the efficient computation of the restricted log likelihood Eq 5 by using low rank update of $\mathbf{Q}$ as follows.

First, the low-rank update of $\mathbf{Q}$ is

$$\begin{aligned}
\mathbf{Q} &= \mathbf{P}^{\mathrm{T}}\mathbf{H}^{-1}\mathbf{P} \\
&= (\mathbf{SHS})^{\dagger} \\
&= \left(\mathbf{S}\left(\mathbf{Z}_{\mathrm{c}}\mathbf{K}_{\mathrm{c}}\mathbf{Z}_{\mathrm{c}}^{\mathrm{T}} + \tilde{\mathbf{W}}_{\mathrm{r}_i}\tilde{\mathbf{\Gamma}}_{\mathrm{r}_i}\tilde{\mathbf{W}}_{\mathrm{r}_i}^{\mathrm{T}} + \mathbf{I}_n\delta_{\mathrm{e}}\right)\mathbf{S}\right)^{\dagger} \\
&= \left(\mathbf{S}\left(\mathbf{Z}_{\mathrm{c}}\mathbf{K}_{\mathrm{c}}\mathbf{Z}_{\mathrm{c}}^{\mathrm{T}} + \mathbf{I}_n\delta_{\mathrm{e}}\right)\mathbf{S} + \mathbf{S}\tilde{\mathbf{W}}_{\mathrm{r}_i}\tilde{\mathbf{\Gamma}}_{\mathrm{r}_i}\tilde{\mathbf{W}}_{\mathrm{r}_i}^{\mathrm{T}}\mathbf{S}\right)^{\dagger} \\
&= \left(\mathbf{U}_{\mathrm{L}}\left(\mathbf{\Lambda}_{\mathrm{c}} + \mathbf{I}_{n-p}\delta_{\mathrm{e}}\right)\mathbf{U}_{\mathrm{L}}^{\mathrm{T}} + \mathbf{U}_{\mathrm{L}}\mathbf{U}_{\mathrm{L}}^{\mathrm{T}}\tilde{\mathbf{W}}_{\mathrm{r}_i}\tilde{\mathbf{\Gamma}}_{\mathrm{r}_i}\tilde{\mathbf{W}}_{\mathrm{r}_i}^{\mathrm{T}}\mathbf{U}_{\mathrm{L}}\mathbf{U}_{\mathrm{L}}^{\mathrm{T}}\right)^{\dagger} \\
&= \left(\mathbf{U}_{\mathrm{L}}\left(\mathbf{\Lambda}_{\mathrm{c}} + \mathbf{I}_{n-p}\delta_{\mathrm{e}} + \mathbf{U}_{\mathrm{L}}^{\mathrm{T}}\tilde{\mathbf{W}}_{\mathrm{r}_i}\tilde{\mathbf{\Gamma}}_{\mathrm{r}_i}\tilde{\mathbf{W}}_{\mathrm{r}_i}^{\mathrm{T}}\mathbf{U}_{\mathrm{L}}\right)\mathbf{U}_{\mathrm{L}}^{\mathrm{T}}\right)^{\dagger} \\
&= \mathbf{U}_{\mathrm{L}}\left(\mathbf{\Lambda}_{\mathrm{c}} + \mathbf{I}_{n-p}\delta_{\mathrm{e}} + \mathbf{U}_{\mathrm{L}}^{\mathrm{T}}\tilde{\mathbf{W}}_{\mathrm{r}_i}\tilde{\mathbf{\Gamma}}_{\mathrm{r}_i}\tilde{\mathbf{W}}_{\mathrm{r}_i}^{\mathrm{T}}\mathbf{U}_{\mathrm{L}}\right)^{-1}\mathbf{U}_{\mathrm{L}}^{\mathrm{T}} \\
&= \mathbf{U}_{\mathrm{L}}\left(\mathbf{\Lambda}_{\mathrm{c}} + \mathbf{I}_{n-p}\delta_{\mathrm{e}}\right)^{-1}\mathbf{U}_{\mathrm{L}}^{\mathrm{T}} \\
&\quad - \mathbf{U}_{\mathrm{L}}\left(\mathbf{\Lambda}_{\mathrm{c}} + \mathbf{I}_{n-p}\delta_{\mathrm{e}}\right)^{-1}\mathbf{U}_{\mathrm{L}}^{\mathrm{T}}\tilde{\mathbf{W}}_{\mathrm{r}_i} \\
&\quad \cdot \left(\tilde{\mathbf{\Gamma}}_{\mathrm{r}_i}^{-1} + \tilde{\mathbf{W}}_{\mathrm{r}_i}^{\mathrm{T}}\mathbf{U}_{\mathrm{L}}\left(\mathbf{\Lambda}_{\mathrm{c}} + \mathbf{I}_{n-p}\delta_{\mathrm{e}}\right)^{-1}\mathbf{U}_{\mathrm{L}}^{\mathrm{T}}\tilde{\mathbf{W}}_{\mathrm{r}_i}\right)^{-1} \\
&\quad \cdot \tilde{\mathbf{W}}_{\mathrm{r}_i}^{\mathrm{T}}\mathbf{U}_{\mathrm{L}}\left(\mathbf{\Lambda}_{\mathrm{c}} + \mathbf{I}_{n-p}\delta_{\mathrm{e}}\right)^{-1}\mathbf{U}_{\mathrm{L}}^{\mathrm{T}} \\
&= \mathbf{O}_{\mathrm{c}} - \mathbf{O}_{\mathrm{c}}\tilde{\mathbf{W}}_{\mathrm{r}_i}\left(\tilde{\mathbf{\Gamma}}_{\mathrm{r}_i}^{-1} + \tilde{\mathbf{W}}_{\mathrm{r}_i}^{\mathrm{T}}\mathbf{O}_{\mathrm{c}}\tilde{\mathbf{W}}_{\mathrm{r}_i}\right)^{-1}\tilde{\mathbf{W}}_{\mathrm{r}_i}^{\mathrm{T}}\mathbf{O}_{\mathrm{c}}
\end{aligned} \tag{7}$$

Here, we use Proposition 6, 5, 2, 7 and the Woodbury identity, and we define the eigen decomposition of $\mathbf{S}\mathbf{Z}_{\mathrm{c}}\mathbf{K}_{\mathrm{c}}\mathbf{Z}_{\mathrm{c}}^{\mathrm{T}}\mathbf{S} = \mathbf{U}_{\mathrm{L}}\mathbf{\Lambda}_{\mathrm{c}}\mathbf{U}_{\mathrm{L}}^{\mathrm{T}}$ where $\mathbf{\Lambda}_{\mathrm{c}}$ is a $n-p \times n-p$ diagonal matrix and $n \times n$ matrix $\mathbf{O}_{\mathrm{c}} = \mathbf{U}_{\mathrm{L}}\left(\mathbf{\Lambda}_{\mathrm{c}} + \mathbf{I}_{n-p}\delta_{\mathrm{e}}\right)^{-1}\mathbf{U}_{\mathrm{L}}^{\mathrm{T}}$ to shorten the notation. Assuming that the eigen decomposition of $\mathbf{S}\left(\mathbf{Z}_{\mathrm{c}}\mathbf{K}_{\mathrm{c}}\mathbf{Z}_{\mathrm{c}}^{\mathrm{T}} + \mathbf{I}_{n-p}\delta_{\mathrm{e}}\right)\mathbf{S}$ has been pre-computed, the additional computation required will be $\mathbf{O}_{\mathrm{c}}\tilde{\mathbf{W}}_{\mathrm{r}_i}$, an $O(n^2k)$ operation.

Update of the squared form $\mathbf{y}^{\mathrm{T}}\mathbf{Q}\mathbf{y}$

Since we know the derivation of the low-rank update of $\mathbf{Q}$ (Eq 7) now, we can plug this into the squared form.

$$\mathbf{y}^{\mathrm{T}}\mathbf{Q}\mathbf{y} = \mathbf{y}^{\mathrm{T}}\mathbf{O}_{\mathrm{c}}\mathbf{y} - \mathbf{y}^{\mathrm{T}}\mathbf{O}_{\mathrm{c}}\tilde{\mathbf{W}}_{\mathrm{r}_i}\left(\tilde{\mathbf{\Gamma}}_{\mathrm{r}_i}^{-1} + \tilde{\mathbf{W}}_{\mathrm{r}_i}^{\mathrm{T}}\mathbf{O}_{\mathrm{c}}\tilde{\mathbf{W}}_{\mathrm{r}_i}\right)^{-1}\tilde{\mathbf{W}}_{\mathrm{r}_i}^{\mathrm{T}}\mathbf{O}_{\mathrm{c}}\mathbf{y} \tag{8}$$

Update of the determinant $\log |\mathbf{Q}|_+$

Since the update matrix is not necessarily positive semi-definite, we have to slightly modify Eq 7 to avoid numerical instability.

$$\begin{aligned}\log |\mathbf{Q}|_+ &= -\log \left|\mathbf{U}_\mathrm{L}^\mathrm{T}\mathbf{H}\mathbf{U}_\mathrm{L}\right| \\ &= -\log \left|\mathbf{I}_{n-p}\delta_\mathrm{e} + \boldsymbol{\Lambda}_\mathrm{c} + \mathbf{U}_\mathrm{L}^\mathrm{T}\tilde{\mathbf{W}}_{\mathrm{r}_i}\tilde{\boldsymbol{\Gamma}}_{\mathrm{r}_i}\tilde{\mathbf{W}}_{\mathrm{r}_i}^\mathrm{T}\mathbf{U}_\mathrm{L}\right| \\ &= -\log \left|\mathbf{I}_{n-p}\delta_\mathrm{e} + \boldsymbol{\Lambda}_\mathrm{c} + \mathbf{A}\mathbf{B}\right| \\ &= -\log \left(\left|\mathbf{I}_{n-p}\delta_\mathrm{e} + \boldsymbol{\Lambda}_\mathrm{c}\right| \cdot \left|\mathbf{I}_{n-p} + \left(\mathbf{I}_{n-p}\delta_\mathrm{e} + \boldsymbol{\Lambda}_\mathrm{c}\right)^{-1}\mathbf{A}\mathbf{B}\right|\right) \\ &= -\log \left|\mathbf{I}_{n-p}\delta_\mathrm{e} + \boldsymbol{\Lambda}_\mathrm{c}\right| - \log \left|\mathbf{I}_k + \mathbf{B}\left(\mathbf{I}_{n-p}\delta_\mathrm{e} + \boldsymbol{\Lambda}_\mathrm{c}\right)^{-1}\mathbf{A}\right| \end{aligned} \tag{9}$$

Here, we used Proposition 5, 6, 7 and the Sylvester's determinant identity, and we also define a $n-p \times k$ matrix $\mathbf{A} = \mathbf{U}_\mathrm{L}^\mathrm{T}\tilde{\mathbf{W}}_{\mathrm{r}_i}\tilde{\boldsymbol{\Gamma}}_{\mathrm{r}_i}$ and a $k \times n-p$ matrix $\mathbf{B} = \tilde{\mathbf{W}}_{\mathrm{r}_i}^\mathrm{T}\mathbf{U}_\mathrm{L}$. When calculating the second term of Eq 9, we have to be careful about the possibility that $\mathbf{I}_k + \mathbf{B}\left(\mathbf{I}_{n-p}\delta_\mathrm{e} + \boldsymbol{\Lambda}_\mathrm{c}\right)^{-1}\mathbf{A}$ is not necessarily positive semi-definite. Since the matrix $\mathbf{Q}$ is positive semi-definite, it is possible to calculate the second term of Eq 9, however, the matrix $\mathbf{I}_k + \mathbf{B}\left(\mathbf{I}_{n-p}\delta_\mathrm{e} + \boldsymbol{\Lambda}_\mathrm{c}\right)^{-1}\mathbf{A}$ has an even numbers of negative eigen values, so instead of simply computing the determinant of this matrix, we have to avoid taking logarithms of such negative eigen values.

Estimation of variance components

Now we can derive the efficient computation of the restricted log likelihood from Eq 5, Eq 8 and Eq 9, we can optimize γ_{r_i} and δ_e over maximazation of Eq 5 by using L-BFGS optimzation method as we introduce in the paper [11].

Then, we calculated the weighted variance-covariance matrix by γ_{r_i}, and reestimate variance components by using EMMA (efficient mixed model association) or GEMMA (genome wide efficient mixed model association) [6, 12].

Discussion on the identity of $\tilde{\mathbf{W}}_{\mathrm{r}_i}$ and $\tilde{\boldsymbol{\Gamma}}_{\mathrm{r}_i}$

In this subsection, we discuss on what $\tilde{\mathbf{W}}_{\mathrm{r}_i}$ and $\tilde{\boldsymbol{\Gamma}}_{\mathrm{r}_i}$ correspond to depending on the kind of the kernels for $\mathbf{K}_{\mathrm{r}_i}$.

For the case where $\mathbf{K}_{\mathrm{r}_i}$ is the linear kernel

Here, we discuss the case where $\mathbf{K}_{\mathrm{r}_i}$ is calculated as the linear kernel of the marker genotype $\mathbf{W}_{\mathrm{r}_i}$ belonging to the i-th SNP-set.

For example, if we assume $\mathbf{K}_{\mathrm{r}_i}$ is the additive genetic matrix of $\mathbf{W}_{\mathrm{r}_i}$, $\tilde{\mathbf{W}}_{\mathrm{r}_i}$ corresponds to

$$\tilde{\mathbf{W}}_{\mathrm{r}_i} = \frac{\mathbf{Z}_{\mathrm{r}_i}\left(\mathbf{W}_{\mathrm{r}_i} + \mathbf{1}_{m_{\mathrm{r}_i} \times M_i} - 2 \cdot \boldsymbol{\Phi}\right)}{2 \cdot \sum_{m=1}^{M_i} p_m \left(1 - p_m\right)}, \tag{10}$$

where p_m is the allele frequency of the 1 allele at marker m and $\boldsymbol{\Phi}$ is a $m_{\mathrm{r}_i} \times M_i$ matrix whose m_th column equals to $\boldsymbol{\Phi} = p_m \cdot \mathbf{1}_{m_{\mathrm{r}_i} \times 1}$ [13].

On the other hand, $\tilde{\boldsymbol{\Gamma}}_{\mathrm{r}_i}$ corresponds to

$$\tilde{\boldsymbol{\Gamma}}_{\mathrm{r}_i} = \mathbf{I}_{M_i}\gamma_{\mathrm{r}_i}, \tag{11}$$

so in this case, $\tilde{\mathbf{\Gamma}}_{\mathrm{r}_i}$ is a $M_i \times M_i$ diagonal matrix. Here, k in Eq 7 equals to the number of SNPs in the i-th SNP-set, M_i.

For the case where $\mathbf{K}_{\mathrm{r}_i}$ is the exponential or gaussian kernel

Here, we discuss the case where $\mathbf{K}_{\mathrm{r}_i}$ is calculated as the exponential or gaussian kernel of the marker genotype $\mathbf{W}_{\mathrm{r}_i}$ belonging to the i-th SNP-set.

First, we calculated the Euclidean distance matrix $\mathbf{D}_{\mathrm{r}_i}$ from the marker genotype $\mathbf{W}_{\mathrm{r}_i}$ belonging to the i-th SNP-set. Then for the case where $\mathbf{K}_{\mathrm{r}_i}$ is the exponential kernel, $\mathbf{K}_{\mathrm{r}_i}$ is calculated as

$$\mathbf{K}_{\mathrm{r}_i} = \exp\left(-\frac{h_{\mathrm{r}_i}\mathbf{D}_{\mathrm{r}_i}}{\sqrt{M_i}}\right), \tag{12}$$

where h_{r_i} is a hyperparameter calculated as the inverse of median of the off-diagonal elements of $\mathbf{D}^2_{\mathrm{r}_i}/M_i$ for the default setting of RAINBOW. To scale the distance matrix, the distance matrix is divided by $\sqrt{M_i}$ in the exponential. Similarly, for the case where $\mathbf{K}_{\mathrm{r}_i}$ is the gaussian kernel, $\mathbf{K}_{\mathrm{r}_i}$ is calculated as

$$\mathbf{K}_{\mathrm{r}_i} = \exp\left(-\frac{h_{\mathrm{r}_i}\mathbf{D}^2_{\mathrm{r}_i}}{M_i}\right), \tag{13}$$

where the term in the exponential is also deivide by M_i to fit its scale to the linear kernel.

In this case, we cannot apply the decomposition of $\mathbf{K}_{\mathrm{r}_i}$ as seen in the linear kernel case, however, it is assumed that the rank of $\mathbf{K}_{\mathrm{r}_i}$ is still much smaller than the number of observations, n. This is because, since there should be strong linkage disequilibrium between SNPs in each SNP-set and many accessions share the same SNPs in that SNP-set, the number of genotypes in that SNP-set m_{r_i} may be much smaller than n. Therefore, in such case,

$$\tilde{\mathbf{W}}_{\mathrm{r}_i} = \mathbf{Z}_{\mathrm{r}_i}, \tag{14}$$

$$\tilde{\mathbf{\Gamma}}_{\mathrm{r}_i} = \mathbf{K}_{\mathrm{r}_i}\gamma_{\mathrm{r}_i} \tag{15}$$

Here, k in Eq 7 equals to the number of SNPs in the genotypes in the i_{th} SNP-set, m_{r_i}. We should be careful about that $\tilde{\mathbf{\Gamma}}_{\mathrm{r}_i}$ is not a diagonal matrix in this case.

Efficient likelihood ratio test for dominance and epistatic effects

In this section, we extend the efficient LR test for the one random effect of each SNP-set to that for multiple random effects of the SNP-set. In this case, the multi-kernel mixed model will be

$$\mathbf{y} = \mathbf{X}\boldsymbol{\beta} + \mathbf{Z}_{\mathrm{c}}\mathbf{u}_{\mathrm{c}} + \sum_{l=1}^{L}\mathbf{Z}_{\mathrm{r}_i,l}\mathbf{u}_{\mathrm{r}_i,l} + \boldsymbol{\epsilon}, \tag{16}$$

where $\mathbf{u}_{\mathrm{r}_i,l}$ is the l_{th} random effect of the i_{th} SNP-set and $\mathbf{Z}_{\mathrm{r}_i,l}$ is a $n \times m_{\mathrm{r}_i,l}$ design matrix which correspond to $\mathbf{u}_{\mathrm{r}_i,l}$. Examples of multiple random effects are additive effects, dominance effects and epistatic effects, and the

test for the significance of these effects will be described later.

The restricted log likelihood is still Eq 5, however, here

$$\begin{aligned}\mathbf{H} &= \mathbf{Z}_{\rm c}\mathbf{K}_{\rm c}\mathbf{Z}_{\rm c}^{\rm T} + \sum_{l=1}^{L}\mathbf{Z}_{{\rm r}_i,l}\mathbf{K}_{{\rm r}_i,l}\mathbf{Z}_{{\rm r}_i,l}^{\rm T}\gamma_{{\rm r}_i,l} + \mathbf{I}_n\delta_{\rm e}\\ &= \mathbf{Z}_{\rm c}\mathbf{K}_{\rm c}\mathbf{Z}_{\rm c}^{\rm T} + \sum_{l=1}^{L}\tilde{\mathbf{W}}_{{\rm r}_i,l}\tilde{\boldsymbol{\Gamma}}_{{\rm r}_i,l}\tilde{\mathbf{W}}_{{\rm r}_i,l}^{\rm T} + \mathbf{I}_n\delta_{\rm e},\end{aligned} \tag{17}$$

where $\mathbf{K}_{{\rm r}_i,l}$, $\gamma_{{\rm r}_i,l}$, $\tilde{\mathbf{W}}_{{\rm r}_i,l}$ and $\tilde{\boldsymbol{\Gamma}}_{{\rm r}_i,l}$ are the extensions of $\mathbf{K}_{{\rm r}_i}$, $\gamma_{{\rm r}_i}$, $\tilde{\mathbf{W}}_{{\rm r}_i}$ and $\tilde{\boldsymbol{\Gamma}}_{{\rm r}_i}$ for the $l_{\rm th}$ random effect.

Low rank update of $\mathbf{Q}^{(L)}$

In this case, the low rank update of $\mathbf{Q}$ is realized by extending Eq 7 to

$$\begin{aligned}\mathbf{Q}^{(L)} &= \mathbf{P}^{\rm T}\mathbf{H}^{-1}\mathbf{P}\\
&= (\mathbf{SHS})^{\dagger}\\
&= \left(\mathbf{S}\left(\mathbf{Z}_{\rm c}\mathbf{K}_{\rm c}\mathbf{Z}_{\rm c}^{\rm T} + \sum_{l=1}^{L}\tilde{\mathbf{W}}_{{\rm r}_i,l}\tilde{\boldsymbol{\Gamma}}_{{\rm r}_i,l}\tilde{\mathbf{W}}_{{\rm r}_i,l}^{\rm T} + \mathbf{I}_n\delta_{\rm e}\right)\mathbf{S}\right)^{\dagger}\\
&= \left(\mathbf{S}\left(\mathbf{Z}_{\rm c}\mathbf{K}_{\rm c}\mathbf{Z}_{\rm c}^{\rm T} + \mathbf{I}_n\delta_{\rm e}\right)\mathbf{S} + \sum_{l=1}^{L}\mathbf{S}\tilde{\mathbf{W}}_{{\rm r}_i,l}\tilde{\boldsymbol{\Gamma}}_{{\rm r}_i,l}\tilde{\mathbf{W}}_{{\rm r}_i,l}^{\rm T}\mathbf{S}\right)^{\dagger}\\
&= \left(\mathbf{U}_{\rm L}\left(\boldsymbol{\Lambda}_{\rm c} + \mathbf{I}_{n-p}\delta_{\rm e}\right)\mathbf{U}_{\rm L}^{\rm T} + \sum_{l=1}^{L}\mathbf{U}_{\rm L}\mathbf{U}_{\rm L}^{\rm T}\tilde{\mathbf{W}}_{{\rm r}_i,l}\tilde{\boldsymbol{\Gamma}}_{{\rm r}_i,l}\tilde{\mathbf{W}}_{{\rm r}_i,l}^{\rm T}\mathbf{U}_{\rm L}\mathbf{U}_{\rm L}^{\rm T}\right)^{\dagger}\\
&= \left(\mathbf{U}_{\rm L}\left(\boldsymbol{\Lambda}_{\rm c} + \mathbf{I}_{n-p}\delta_{\rm e} + \sum_{l=1}^{L}\mathbf{U}_{\rm L}^{\rm T}\tilde{\mathbf{W}}_{{\rm r}_i,l}\tilde{\boldsymbol{\Gamma}}_{{\rm r}_i,l}\tilde{\mathbf{W}}_{{\rm r}_i,l}^{\rm T}\mathbf{U}_{\rm L}\right)\mathbf{U}_{\rm L}^{\rm T}\right)^{\dagger}\\
&= \mathbf{U}_{\rm L}\left(\boldsymbol{\Lambda}_{\rm c} + \mathbf{I}_{n-p}\delta_{\rm e} + \sum_{l=1}^{L}\mathbf{U}_{\rm L}^{\rm T}\tilde{\mathbf{W}}_{{\rm r}_i,l}\tilde{\boldsymbol{\Gamma}}_{{\rm r}_i,l}\tilde{\mathbf{W}}_{{\rm r}_i,l}^{\rm T}\mathbf{U}_{\rm L}\right)^{-1}\mathbf{U}_{\rm L}^{\rm T}\\
&= \mathbf{U}_{\rm L}\left(\boldsymbol{\Lambda}_{\rm c} + \mathbf{I}_{n-p}\delta_{\rm e} + \sum_{l=1}^{L-1}\tilde{\mathbf{W}}_{{\rm r}_i,l}\tilde{\boldsymbol{\Gamma}}_{{\rm r}_i,l}\tilde{\mathbf{W}}_{{\rm r}_i,l}^{\rm T}\right)^{-1}\mathbf{U}_{\rm L}^{\rm T}\\
&\quad - \mathbf{U}_{\rm L}\left(\boldsymbol{\Lambda}_{\rm c} + \mathbf{I}_{n-p}\delta_{\rm e} + \sum_{l=1}^{L-1}\tilde{\mathbf{W}}_{{\rm r}_i,l}\tilde{\boldsymbol{\Gamma}}_{{\rm r}_i,l}\tilde{\mathbf{W}}_{{\rm r}_i,l}^{\rm T}\right)^{-1}\mathbf{U}_{\rm L}^{\rm T}\tilde{\mathbf{W}}_{{\rm r}_i,L}\\
&\quad \cdot\left(\tilde{\boldsymbol{\Gamma}}_{{\rm r}_i,L}^{-1} + \tilde{\mathbf{W}}_{{\rm r}_i,L}^{\rm T}\mathbf{U}_{\rm L}\left(\boldsymbol{\Lambda}_{\rm c} + \mathbf{I}_{n-p}\delta_{\rm e} + \sum_{l=1}^{L-1}\tilde{\mathbf{W}}_{{\rm r}_i,l}\tilde{\boldsymbol{\Gamma}}_{{\rm r}_i,l}\tilde{\mathbf{W}}_{{\rm r}_i,l}^{\rm T}\right)^{-1}\mathbf{U}_{\rm L}^{\rm T}\tilde{\mathbf{W}}_{{\rm r}_i,L}\right)^{-1}\\
&\quad \cdot\tilde{\mathbf{W}}_{{\rm r}_i,L}^{\rm T}\mathbf{U}_{\rm L}\left(\boldsymbol{\Lambda}_{\rm c} + \mathbf{I}_{n-p}\delta_{\rm e} + \sum_{l=1}^{L-1}\tilde{\mathbf{W}}_{{\rm r}_i,l}\tilde{\boldsymbol{\Gamma}}_{{\rm r}_i,l}\tilde{\mathbf{W}}_{{\rm r}_i,l}^{\rm T}\right)^{-1}\mathbf{U}_{\rm L}^{\rm T}\\
&= \mathbf{Q}^{(L-1)} - \mathbf{Q}^{(L-1)}\tilde{\mathbf{W}}_{{\rm r}_i,L}\left(\tilde{\boldsymbol{\Gamma}}_{{\rm r}_i,L}^{-1} + \tilde{\mathbf{W}}_{{\rm r}_i,L}^{\rm T}\mathbf{Q}^{(L-1)}\tilde{\mathbf{W}}_{{\rm r}_i,L}\right)^{-1}\tilde{\mathbf{W}}_{{\rm r}_i,L}^{\rm T}\mathbf{Q}^{(L-1)}\end{aligned} \tag{18}$$

Here, we use Proposition 6, 5, 2, 7 and the Woodbury identity, and we define $\mathbf{Q}^{(L)}$ as the $\mathbf{Q}$ matrix for the L random effects of the SNP-set, so

$\mathbf{Q}^{(L)} = \mathbf{U}_\mathrm{L}\left(\mathbf{\Lambda}_\mathrm{c} + \mathbf{I}_{n-p}\delta_\mathrm{e} + \sum_{l=1}^{L}\mathbf{U}_\mathrm{L}^\mathrm{T}\tilde{\mathbf{W}}_{\mathrm{r}_i,l}\tilde{\mathbf{\Gamma}}_{\mathrm{r}_i,l}\tilde{\mathbf{W}}_{\mathrm{r}_i,l}^\mathrm{T}\mathbf{U}_\mathrm{L}\right)^{-1}\mathbf{U}_\mathrm{L}$. To compute $\mathbf{Q}^{(L)}$, we set $\mathbf{Q}^{(0)} = \mathbf{U}_\mathrm{L}\left(\mathbf{\Lambda}_\mathrm{c} + \mathbf{I}_{n-p}\delta_\mathrm{e}\right)$ first, and repeat Eq 18 for L steps. The computation of $\mathbf{Q}^{(L)}$ will be an $O(n^2\sum_{l=1}^{L}k_l)$ operation and it is much efficient if all $k_l \ll n$ where k_l is the rank of $\tilde{\mathbf{W}}_{\mathrm{r}_i,l}$.

Update of the determinant $\log\left|\mathbf{Q}^{(L)}\right|_+$

We also extend Eq 9 to

$$\begin{aligned}
\log\left|\mathbf{Q}^{(L)}\right|_+ &= -\log\left|\mathbf{U}_\mathrm{L}^\mathrm{T}\mathbf{H}\mathbf{U}_\mathrm{L}\right| \\
&= -\log\left|\mathbf{I}_{n-p}\delta_\mathrm{e} + \mathbf{\Lambda}_\mathrm{c} + \sum_{l=1}^{L}\mathbf{U}_\mathrm{L}^\mathrm{T}\tilde{\mathbf{W}}_{\mathrm{r}_i,l}\tilde{\mathbf{\Gamma}}_{\mathrm{r}_i,l}\tilde{\mathbf{W}}_{\mathrm{r}_i,l}^\mathrm{T}\mathbf{U}_\mathrm{L}\right| \\
&= -\log\left|\mathbf{I}_{n-p}\delta_\mathrm{e} + \mathbf{\Lambda}_\mathrm{c} + \sum_{l=1}^{L-1}\mathbf{A}^{(l)}\mathbf{B}^{(l)} + \mathbf{A}^{(L)}\mathbf{B}^{(L)}\right| \\
&= -\log\left|\mathbf{I}_{n-p}\delta_\mathrm{e} + \mathbf{\Lambda}_\mathrm{c} + \sum_{l=1}^{L-1}\mathbf{A}^{(l)}\mathbf{B}^{(l)}\right| \\
&\quad -\log\left|\mathbf{I}_{n-p} + \left(\mathbf{I}_{n-p}\delta_\mathrm{e} + \mathbf{\Lambda}_\mathrm{c} + \sum_{l=1}^{L-1}\mathbf{A}^{(l)}\mathbf{B}^{(l)}\right)^{-1}\mathbf{A}^{(L)}\mathbf{B}^{(L)}\right| \\
&= \log\left|\mathbf{Q}^{(L-1)}\right|_+ - \log\left|\mathbf{I}_{k_L} + \mathbf{B}^{(L)}\mathbf{Q}^{(L-1)}\mathbf{A}^{(L)}\right| \qquad (19)
\end{aligned}$$

Here, we used Proposition 5, 6, 7 and the Sylvester's determinant identity, and we also define a $n-p\times k_L$ matrix $\mathbf{A}^{(L)} = \mathbf{U}_\mathrm{L}^\mathrm{T}\tilde{\mathbf{W}}_{\mathrm{r}_i,L}\tilde{\mathbf{\Gamma}}_{\mathrm{r}_i,L}$ and a $k\times n-p$ matrix $\mathbf{B}^{(L)} = \tilde{\mathbf{W}}_{\mathrm{r}_i,L}^\mathrm{T}\mathbf{U}_\mathrm{L}$. Computation of $\log\left|\mathbf{Q}^{(L)}\right|_+$ can be realized by repeating Eq 19 after setting $\log\left|\mathbf{Q}^{(0)}\right|_+ = -\log\left|\mathbf{I}_{n-p}\delta_\mathrm{e} + \mathbf{\Lambda}_\mathrm{c}\right|$. Here, when calculating the second term of Eq 19, the notes mentioned for Eq 9 still exists in this case.

The LR test for dominance and epistatic effects

To test the dominance or epistatic effects of each SNP-set, it should be assumed that the term of additive effects of each SNP-set is included both in the null model and the alternative model. Therefore we should assume the model such as Eq 16 for the alternative model in this case.

If we define $\mathbf{K}_{\mathrm{r}_i,\mathrm{d}}$ is the dominance genetic matrix of $\mathbf{W}_{\mathrm{r}_i}$, $\tilde{\mathbf{W}}_{\mathrm{r}_i,\mathrm{d}}$ ($\tilde{\mathbf{W}}_{\mathrm{r}_i}$ for the dominance effects) corresponds to

$$\tilde{\mathbf{W}}_{\mathrm{r}_i,\mathrm{d}} = \frac{\mathbf{Z}_{\mathrm{r}_i}\left(\mathbf{1}_{m_{\mathrm{r}_i}\times M_i} - ||\mathbf{W}_{\mathrm{r}_i}||\right)}{2\cdot\sum_{m=1}^{M_i}p_m\left(1-p_m\right)\left(1-p_m\left(1-p_m\right)\right)}, \qquad (20)$$

and $\tilde{\mathbf{\Gamma}}_{\mathrm{r}_i,\mathrm{d}}$ is

$$\tilde{\mathbf{\Gamma}}_{\mathrm{r}_i,\mathrm{d}} = \mathbf{I}_{M_i}\gamma_{\mathrm{r}_i,\mathrm{d}}, \qquad (21)$$

where $\gamma_{\mathrm{r}_i,\mathrm{d}}$ is the weight for the dominance effect of i_th SNP-set to be estimated [14]. Therefore, to test the significance of dominance effects, compare the restricted log likelihood of the model including both additive and dominance effects such as Eq 16 ($l = 2$) with that of the model including only additive effects such as Eq 1.

For the random effects of epistatic effects between two SNP-sets consisting of the same number of SNPs M, $\tilde{\mathbf{W}}_{\mathrm{r}_{ij},\mathrm{aa}}$, $\tilde{\mathbf{W}}_{\mathrm{r}_{ij},\mathrm{ad}}$, $\tilde{\mathbf{W}}_{\mathrm{r}_{ij},\mathrm{da}}$ and $\tilde{\mathbf{W}}_{\mathrm{r}_{ij},\mathrm{dd}}$($\tilde{\mathbf{W}}_{\mathrm{r}_{ij}}$ for additive $\times$ additive, additive $\times$ dominance, dominance $\times$ additive and dominance $\times$ dominance epistatic effects between the i_{th} and the j_{th} SNP-sets of interest respectively) correspond to

$$\tilde{\mathbf{W}}_{\mathrm{r}_{ij},\mathrm{aa}} = \tilde{\mathbf{W}}_{\mathrm{r}_i,\mathrm{a}} \circ \tilde{\mathbf{W}}_{\mathrm{r}_j,\mathrm{a}}, \tag{22}$$
$$\tilde{\mathbf{W}}_{\mathrm{r}_{ij},\mathrm{ad}} = \tilde{\mathbf{W}}_{\mathrm{r}_i,\mathrm{a}} \circ \tilde{\mathbf{W}}_{\mathrm{r}_j,\mathrm{d}}, \tag{23}$$
$$\tilde{\mathbf{W}}_{\mathrm{r}_{ij},\mathrm{da}} = \tilde{\mathbf{W}}_{\mathrm{r}_i,\mathrm{d}} \circ \tilde{\mathbf{W}}_{\mathrm{r}_j,\mathrm{a}}, \tag{24}$$
$$\tilde{\mathbf{W}}_{\mathrm{r}_{ij},\mathrm{dd}} = \tilde{\mathbf{W}}_{\mathrm{r}_i,\mathrm{d}} \circ \tilde{\mathbf{W}}_{\mathrm{r}_j,\mathrm{d}}, \tag{25}$$

where $\tilde{\mathbf{W}}_{\mathrm{r}_i,\mathrm{a}}$ is defined by Eq 10 and $\tilde{\mathbf{W}}_{\mathrm{r}_j,\mathrm{d}}$ is defined by Eq 20 [15, 14]. On the other hand, correponding $\tilde{\mathbf{\Gamma}}_{\mathrm{r}_{ij},\mathrm{aa}}$, $\tilde{\mathbf{\Gamma}}_{\mathrm{r}_{ij},\mathrm{ad}}$, $\tilde{\mathbf{\Gamma}}_{\mathrm{r}_{ij},\mathrm{da}}$ and $\tilde{\mathbf{\Gamma}}_{\mathrm{r}_{ij},\mathrm{dd}}$ are

$$\tilde{\mathbf{\Gamma}}_{\mathrm{r}_{ij},\mathrm{aa}} = \mathbf{I}_M \gamma_{\mathrm{r}_{ij},\mathrm{aa}}, \tag{26}$$
$$\tilde{\mathbf{\Gamma}}_{\mathrm{r}_{ij},\mathrm{ad}} = \mathbf{I}_M \gamma_{\mathrm{r}_{ij},\mathrm{ad}}, \tag{27}$$
$$\tilde{\mathbf{\Gamma}}_{\mathrm{r}_{ij},\mathrm{da}} = \mathbf{I}_M \gamma_{\mathrm{r}_{ij},\mathrm{da}}, \tag{28}$$
$$\tilde{\mathbf{\Gamma}}_{\mathrm{r}_{ij},\mathrm{dd}} = \mathbf{I}_M \gamma_{\mathrm{r}_{ij},\mathrm{dd}}, \tag{29}$$

where $\gamma_{\mathrm{r}_{ij},\mathrm{aa}}$, $\gamma_{\mathrm{r}_{ij},\mathrm{ad}}$, $\gamma_{\mathrm{r}_{ij},\mathrm{da}}$ and $\gamma_{\mathrm{r}_{ij},\mathrm{dd}}$ are weights for each epistatic effects to be estimated. In this case, to test the significance of these epistatic effects, compare the restricted log likelihood of the model including additive and dominance effects of two SNP-set and four epistatic effects such as Eq 16 ($l = 8$) with that of the model including only additive and dominance effects of two SNP-set such as Eq 16 (l = 4).

Propositions

Proposition 1. $\mathbf{SX} = \mathbf{0}$, $\mathbf{PX} = \mathbf{0}$ *and* $\mathbf{U}_{\mathrm{L}}^{\mathrm{T}}\mathbf{X} = \mathbf{0}$.

Proof.

$$\mathbf{SX} = \mathbf{X} - \mathbf{X}\left(\mathbf{X}^{\mathrm{T}}\mathbf{X}\right)^{-1}\mathbf{X}^{\mathrm{T}}\mathbf{X} = \mathbf{0},$$
$$\mathbf{PX} = \mathbf{X} - \mathbf{X}\left(\mathbf{X}^{\mathrm{T}}\mathbf{H}^{-1}\mathbf{X}\right)^{-1}\mathbf{X}^{\mathrm{T}}\mathbf{H}^{-1}\mathbf{X} = \mathbf{0},$$

and because $\mathbf{PX} = \mathbf{0}$, $\mathbf{P}_{\mathrm{L}}\mathbf{X} = \mathbf{0}$. Therefore, $\mathbf{U}_{\mathrm{L}}^{\mathrm{T}}\mathbf{X} = \mathbf{0}$. □

Proposition 2. $\mathbf{S}^2 = \mathbf{S}$.

Proof.

$$\begin{aligned}
\mathbf{S}^2 &= \left(\mathbf{I}_n - \mathbf{X}\left(\mathbf{X}^{\mathrm{T}}\mathbf{X}\right)^{-1}\mathbf{X}^{\mathrm{T}}\right)\left(\mathbf{I}_n - \mathbf{X}\left(\mathbf{X}^{\mathrm{T}}\mathbf{X}\right)^{-1}\mathbf{X}^{\mathrm{T}}\right), \\
&= \mathbf{I}_n - 2\cdot\mathbf{X}\left(\mathbf{X}^{\mathrm{T}}\mathbf{X}\right)^{-1}\mathbf{X}^{\mathrm{T}} + \mathbf{X}\left(\mathbf{X}^{\mathrm{T}}\mathbf{X}\right)^{-1}\mathbf{X}^{\mathrm{T}}\mathbf{X}\left(\mathbf{X}^{\mathrm{T}}\mathbf{X}\right)^{-1}\mathbf{X}^{\mathrm{T}} \\
&= \mathbf{I}_n - \mathbf{X}\left(\mathbf{X}^{\mathrm{T}}\mathbf{X}\right)^{-1}\mathbf{X}^{\mathrm{T}} \\
&= \mathbf{S}
\end{aligned}$$

This characteristic is called idempotent. □

Proposition 3. $\mathbf{PS} = \mathbf{P}$ *and* $\mathbf{SP} = \mathbf{S}$.

Proof.

$$\begin{aligned}
\mathbf{PS} &= \left(\mathbf{I}_n - \mathbf{X}\left(\mathbf{X}^{\mathrm{T}}\mathbf{V}^{-1}\mathbf{X}\right)^{-1}\mathbf{X}^{\mathrm{T}}\mathbf{V}^{-1}\right)\left(\mathbf{I}_n - \mathbf{X}\left(\mathbf{X}^{\mathrm{T}}\mathbf{X}\right)^{-1}\mathbf{X}^{\mathrm{T}}\right) \\
&= \mathbf{I}_n - \mathbf{X}\left(\mathbf{X}^{\mathrm{T}}\mathbf{V}^{-1}\mathbf{X}\right)^{-1}\mathbf{X}^{\mathrm{T}}\mathbf{V}^{-1} - \mathbf{X}\left(\mathbf{X}^{\mathrm{T}}\mathbf{X}\right)^{-1}\mathbf{X}^{\mathrm{T}} \\
&\quad + \mathbf{X}\left(\mathbf{X}^{\mathrm{T}}\mathbf{V}^{-1}\mathbf{X}\right)^{-1}\mathbf{X}^{\mathrm{T}}\mathbf{V}^{-1}\mathbf{X}\left(\mathbf{X}^{\mathrm{T}}\mathbf{X}\right)^{-1}\mathbf{X}^{\mathrm{T}} \\
&= \mathbf{I}_n - \mathbf{X}\left(\mathbf{X}^{\mathrm{T}}\mathbf{V}^{-1}\mathbf{X}\right)^{-1}\mathbf{X}^{\mathrm{T}}\mathbf{V}^{-1} \\
&= \mathbf{P},
\end{aligned}$$

and

$$\begin{aligned}
\mathbf{SP} &= \left(\mathbf{I}_n - \mathbf{X}\left(\mathbf{X}^{\mathrm{T}}\mathbf{X}\right)^{-1}\mathbf{X}^{\mathrm{T}}\right)\left(\mathbf{I}_n - \mathbf{X}\left(\mathbf{X}^{\mathrm{T}}\mathbf{V}^{-1}\mathbf{X}\right)^{-1}\mathbf{X}^{\mathrm{T}}\mathbf{V}^{-1}\right) \\
&= \mathbf{I}_n - \mathbf{X}\left(\mathbf{X}^{\mathrm{T}}\mathbf{V}^{-1}\mathbf{X}\right)^{-1}\mathbf{X}^{\mathrm{T}}\mathbf{V}^{-1} - \mathbf{X}\left(\mathbf{X}^{\mathrm{T}}\mathbf{X}\right)^{-1}\mathbf{X}^{\mathrm{T}} \\
&\quad + \mathbf{X}\left(\mathbf{X}^{\mathrm{T}}\mathbf{X}\right)^{-1}\mathbf{X}^{\mathrm{T}}\mathbf{X}\left(\mathbf{X}^{\mathrm{T}}\mathbf{V}^{-1}\mathbf{X}\right)^{-1}\mathbf{X}^{\mathrm{T}}\mathbf{V}^{-1} \\
&= \mathbf{I}_n - \mathbf{X}\left(\mathbf{X}^{\mathrm{T}}\mathbf{X}\right)^{-1}\mathbf{X}^{\mathrm{T}} \\
&= \mathbf{S}
\end{aligned}$$

□

Proposition 4. $\mathbf{P}^2 = \mathbf{P}$.

Proof.

$$\begin{aligned}
\mathbf{P}^2 &= \left(\mathbf{I}_n - \mathbf{X}\left(\mathbf{X}^{\mathrm{T}}\mathbf{V}^{-1}\mathbf{X}\right)^{-1}\mathbf{X}^{\mathrm{T}}\mathbf{V}^{-1}\right)\left(\mathbf{I}_n - \mathbf{X}\left(\mathbf{X}^{\mathrm{T}}\mathbf{V}^{-1}\mathbf{X}\right)^{-1}\mathbf{X}^{\mathrm{T}}\mathbf{V}^{-1}\right) \\
&= \mathbf{I}_n - 2\times\mathbf{X}\left(\mathbf{X}^{\mathrm{T}}\mathbf{V}^{-1}\mathbf{X}\right)^{-1}\mathbf{X}^{\mathrm{T}}\mathbf{V}^{-1} \\
&\quad + \mathbf{X}\left(\mathbf{X}^{\mathrm{T}}\mathbf{V}^{-1}\mathbf{X}\right)^{-1}\mathbf{X}^{\mathrm{T}}\mathbf{V}^{-1}\mathbf{X}\left(\mathbf{X}^{\mathrm{T}}\mathbf{V}^{-1}\mathbf{X}\right)^{-1}\mathbf{X}^{\mathrm{T}}\mathbf{V}^{-1} \\
&= \mathbf{I}_n - \mathbf{X}\left(\mathbf{X}^{\mathrm{T}}\mathbf{V}^{-1}\mathbf{X}\right)^{-1}\mathbf{X}^{\mathrm{T}}\mathbf{V}^{-1} \\
&= \mathbf{P}
\end{aligned}$$

□

Proposition 5. $\mathbf{P}^{\mathrm{T}}\mathbf{H}^{-1}\mathbf{P} = (\mathbf{SHS})^{\dagger}$.

Proof.
From Proposition 3 and 4,

$$\begin{aligned}(\mathbf{SHS})\left(\mathbf{P}^{\mathrm{T}}\mathbf{H}^{-1}\mathbf{P}\right)(\mathbf{SHS}) &= \mathbf{SHP}^{\mathrm{T}}\mathbf{H}^{-1}\mathbf{PHS} \\ &= \mathbf{SPPHS} \\ &= \mathbf{SHS},\end{aligned}$$

and

$$\begin{aligned}\left(\mathbf{P}^{\mathrm{T}}\mathbf{H}^{-1}\mathbf{P}\right)(\mathbf{SHS})\left(\mathbf{P}^{\mathrm{T}}\mathbf{H}^{-1}\mathbf{P}\right) &= \mathbf{P}^{\mathrm{T}}\mathbf{H}^{-1}\mathbf{PHP}^{\mathrm{T}}\mathbf{H}^{-1}\mathbf{P} \\ &= \mathbf{P}^{\mathrm{T}}\mathbf{H}^{-1}\mathbf{PPP} \\ &= \mathbf{P}^{\mathrm{T}}\mathbf{H}^{-1}\mathbf{P}.\end{aligned}$$

Moreover, the product of $\mathbf{P}^{\mathrm{T}}\mathbf{H}^{-1}\mathbf{P}$ and $\mathbf{SHS}$ is commutative and becomes a Hermitian (in this case, real symmetric) matrix.

$$(\mathbf{SHS})\left(\mathbf{P}^{\mathrm{T}}\mathbf{H}^{-1}\mathbf{P}\right) = \left(\mathbf{P}^{\mathrm{T}}\mathbf{H}^{-1}\mathbf{P}\right)(\mathbf{SHS}) = \mathbf{S}$$

Therefore, from the definition of Moore-Penrose peudo-inverse matrix,

$$\mathbf{P}^{\mathrm{T}}\mathbf{H}^{-1}\mathbf{P} = (\mathbf{SHS})^{\dagger} = \mathbf{U}_{\mathrm{L}}\mathbf{\Lambda}_{L}^{-1}\mathbf{U}_{\mathrm{L}}^{\mathrm{T}}$$

□

Proposition 6. $\mathbf{Q} = \mathbf{P}^{\mathrm{T}}\mathbf{H}^{-1}\mathbf{P}$.

Proof.
By using Proposition 4,

$$\mathbf{P}^{\mathrm{T}}\mathbf{H}^{-1}\mathbf{P} = \mathbf{H}^{-1}\mathbf{PP} = \mathbf{H}^{-1}\mathbf{P} = \mathbf{Q}$$

□

Proposition 7. $\mathbf{U}_{\mathrm{L}}\mathbf{U}_{\mathrm{L}}^{\mathrm{T}} = \mathbf{S}$.

Proof.
By using Proposition 5,

$$\begin{aligned}\mathbf{S} &= (\mathbf{SHS})\left(\mathbf{P}^{\mathrm{T}}\mathbf{H}^{-1}\mathbf{P}\right) = (\mathbf{SHS})(\mathbf{SHS})^{\dagger} \\ &= \mathbf{U}_{\mathrm{L}}\mathbf{\Lambda}_{L}\mathbf{U}_{\mathrm{L}}^{\mathrm{T}}\mathbf{U}_{\mathrm{L}}\mathbf{\Lambda}_{L}^{-1}\mathbf{U}_{\mathrm{L}}^{\mathrm{T}} \\ &= \mathbf{U}_{\mathrm{L}}\mathbf{U}_{\mathrm{L}}^{\mathrm{T}}\end{aligned}$$

□

Proposition 8. $\log\left|\mathbf{P}_{\mathrm{L}}\mathbf{V}\mathbf{P}_{\mathrm{L}}^{\mathrm{T}}\right| = -\log\left|\mathbf{P}^{\mathrm{T}}\mathbf{V}^{-1}\mathbf{P}\right|_{+}$.

Proof.
First,

$$\mathbf{P}_{\mathrm{L}}\mathbf{V}\mathbf{P}_{\mathrm{L}}^{\mathrm{T}} = \mathbf{U}_{\mathrm{L}}^{\mathrm{T}}\mathbf{H}\mathbf{U}_{\mathrm{L}}\sigma_{\mathrm{c}}^{2} = \mathbf{\Lambda}_{L}\sigma_{\mathrm{c}}^{2},$$

because

$$\mathbf{U}_{\mathrm{L}}\mathbf{U}_{\mathrm{L}}^{\mathrm{T}}\mathbf{H}\mathbf{U}_{\mathrm{L}}\mathbf{U}_{\mathrm{L}}^{\mathrm{T}} = \mathbf{SHS} = \mathbf{U}_{\mathrm{L}}\mathbf{\Lambda}_{L}\mathbf{U}_{\mathrm{L}}^{\mathrm{T}},$$

from Proposition 7. Then, by using Proposition 5

$$\begin{aligned}\log|\mathbf{\Lambda}_{L}| &= -\log\left|\mathbf{\Lambda}_{L}^{-1}\right| \\ &= -\log\left|\mathbf{U}_{\mathrm{L}}\mathbf{\Lambda}_{L}^{-1}\mathbf{U}_{\mathrm{L}}^{\mathrm{T}}\right|_{+} \\ &= -\log\left|\mathbf{P}^{\mathrm{T}}\mathbf{H}^{-1}\mathbf{P}\right|_{+}\end{aligned}$$

Therefore,

$$\log\left|\mathbf{P}_{\mathrm{L}}\mathbf{V}\mathbf{P}_{\mathrm{L}}^{\mathrm{T}}\right| = \log\left|\mathbf{\Lambda}_{L}\sigma_{\mathrm{c}}^{2}\right| = -\log\left|\mathbf{P}^{\mathrm{T}}\mathbf{V}^{-1}\mathbf{P}\right|_{+}$$

□

Proposition 9. $\mathbf{P}_{\mathrm{L}}^{\mathrm{T}}\left(\mathbf{P}_{\mathrm{L}}\mathbf{V}\mathbf{P}_{\mathrm{L}}^{\mathrm{T}}\right)^{-1}\mathbf{P}_{\mathrm{L}} = \mathbf{V}^{-1}\mathbf{P}$.

Proof. By using Proposition 8 and 6,

$$\begin{aligned}\mathbf{P}_{\mathrm{L}}^{\mathrm{T}}\left(\mathbf{P}_{\mathrm{L}}\mathbf{V}\mathbf{P}_{\mathrm{L}}^{\mathrm{T}}\right)^{-1}\mathbf{P}_{\mathrm{L}} &= \mathbf{U}_{\mathrm{L}}\left(\mathbf{U}_{\mathrm{L}}^{\mathrm{T}}\mathbf{V}\mathbf{U}_{\mathrm{L}}\right)^{-1}\mathbf{U}_{\mathrm{L}}^{\mathrm{T}} \\ &= \mathbf{U}_{\mathrm{L}}\left(\mathbf{\Lambda}_{L}\sigma_{\mathrm{c}}^{2}\right)^{-1}\mathbf{U}_{\mathrm{L}}^{\mathrm{T}} \\ &= \mathbf{P}^{\mathrm{T}}\mathbf{H}^{-1}\mathbf{P}/\sigma_{\mathrm{c}}^{2} \\ &= \mathbf{Q}/\sigma_{\mathrm{c}}^{2} = \mathbf{V}^{-1}\mathbf{P}\end{aligned}$$

□

Proposition 10. $\frac{1}{2}\log|\mathbf{Q}|_{+} = -\frac{1}{2}\log|\mathbf{H}| + \frac{1}{2}\log\left|\mathbf{X}^{\mathrm{T}}\mathbf{X}\right| - \frac{1}{2}\log\left|\mathbf{X}^{\mathrm{T}}\mathbf{H}^{-1}\mathbf{X}\right|$.

Proof. By using Proposition 6, 5 and 7,

$$
\begin{aligned}
|\mathbf{Q}|_+ &= \left|\mathbf{P}^{\mathrm{T}}\mathbf{H}^{-1}\mathbf{P}\right|_+ \\
&= |\mathbf{SHS}|_+^{-1} \\
&= |\mathbf{\Lambda}_L|^{-1} \cdot \left|\mathbf{X}^{\mathrm{T}}\mathbf{H}^{-1}\mathbf{X}\right| \cdot \left|\mathbf{X}^{\mathrm{T}}\mathbf{H}^{-1}\mathbf{X}\right|^{-1} \\
&= \left|\mathbf{U}_{\mathrm{L}}^{\mathrm{T}}\mathbf{H}\mathbf{U}_{\mathrm{L}}\right|^{-1} \cdot \left|\left(\mathbf{X}^{\mathrm{T}}\mathbf{H}^{-1}\mathbf{X}\right)^{-1}\mathbf{X}^{\mathrm{T}}\mathbf{H}^{-1}\mathbf{X}\left(\mathbf{X}^{\mathrm{T}}\mathbf{H}^{-1}\mathbf{X}\right)^{-1}\right|^{-1} \cdot \left|\mathbf{X}^{\mathrm{T}}\mathbf{H}^{-1}\mathbf{X}\right|^{-1} \\
&= \left|\mathbf{U}_{\mathrm{L}}^{\mathrm{T}}\mathbf{H}\mathbf{U}_{\mathrm{L}}\right|^{-1} \cdot \left|\left(\mathbf{X}^{\mathrm{T}}\mathbf{H}^{-1}\mathbf{X}\right)^{-1}\mathbf{X}^{\mathrm{T}}\mathbf{H}^{-1}\mathbf{H}\mathbf{H}^{-1}\mathbf{X}\left(\mathbf{X}^{\mathrm{T}}\mathbf{H}^{-1}\mathbf{X}\right)^{-1}\right|^{-1} \cdot \left|\mathbf{X}^{\mathrm{T}}\mathbf{H}^{-1}\mathbf{X}\right|^{-1} \\
&= \left|\begin{bmatrix}\mathbf{U}_{\mathrm{L}}^{\mathrm{T}}\mathbf{H}\mathbf{U}_{\mathrm{L}} & \mathbf{0} \\ \mathbf{0} & \left(\mathbf{X}^{\mathrm{T}}\mathbf{H}^{-1}\mathbf{X}\right)^{-1}\mathbf{X}^{\mathrm{T}}\mathbf{H}^{-1}\mathbf{H}\mathbf{H}^{-1}\mathbf{X}\left(\mathbf{X}^{\mathrm{T}}\mathbf{H}^{-1}\mathbf{X}\right)^{-1}\end{bmatrix}\right|^{-1} \cdot \left|\mathbf{X}^{\mathrm{T}}\mathbf{H}^{-1}\mathbf{X}\right|^{-1}
\end{aligned}
$$

Here, we define $\mathbf{M} = \left(\mathbf{X}^{\mathrm{T}}\mathbf{H}^{-1}\mathbf{X}\right)^{-1}\mathbf{X}^{\mathrm{T}}\mathbf{H}^{-1}$ to shorten the notation.

$$
= \left|\begin{bmatrix}\mathbf{U}_{\mathrm{L}}^{\mathrm{T}}\mathbf{H}\mathbf{U}_{\mathrm{L}} & \mathbf{U}_{\mathrm{L}}^{\mathrm{T}}\mathbf{H}\mathbf{M}^{\mathrm{T}} \\ \mathbf{M}\mathbf{H}\mathbf{U}_{\mathrm{L}} & \mathbf{M}\mathbf{H}\mathbf{M}^{\mathrm{T}}\end{bmatrix}\right|^{-1} \cdot \left|\mathbf{X}^{\mathrm{T}}\mathbf{H}^{-1}\mathbf{X}\right|^{-1}
$$

$\mathbf{M}\mathbf{H}\mathbf{U}_{\mathrm{L}} = \mathbf{0}$ because $\mathbf{X}^{\mathrm{T}}\mathbf{U}_{\mathrm{L}} = \mathbf{0}$ (Proposition 1).

$$
\begin{aligned}
&= \left|\begin{bmatrix}\mathbf{U}_{\mathrm{L}}^{\mathrm{T}} \\ \mathbf{M}\end{bmatrix}\mathbf{H}\begin{bmatrix}\mathbf{U}_{\mathrm{L}}^{\mathrm{T}} \\ \mathbf{M}\end{bmatrix}^{\mathrm{T}}\right|^{-1} \cdot \left|\mathbf{X}^{\mathrm{T}}\mathbf{H}^{-1}\mathbf{X}\right|^{-1} \\
&= |\mathbf{H}|^{-1} \cdot \left|\begin{bmatrix}\mathbf{U}_{\mathrm{L}}^{\mathrm{T}} \\ \mathbf{M}\end{bmatrix}\begin{bmatrix}\mathbf{U}_{\mathrm{L}}^{\mathrm{T}} \\ \mathbf{M}\end{bmatrix}^{\mathrm{T}}\right|^{-1} \cdot \left|\mathbf{X}^{\mathrm{T}}\mathbf{H}^{-1}\mathbf{X}\right|^{-1} \\
&= |\mathbf{H}|^{-1} \cdot \left|\begin{bmatrix}\mathbf{U}_{\mathrm{L}}^{\mathrm{T}}\mathbf{U}_{\mathrm{L}} & \mathbf{U}_{\mathrm{L}}^{\mathrm{T}}\mathbf{M}^{\mathrm{T}} \\ \mathbf{M}\mathbf{U}_{\mathrm{L}} & \mathbf{M}\mathbf{M}^{\mathrm{T}}\end{bmatrix}\right|^{-1} \cdot \left|\mathbf{X}^{\mathrm{T}}\mathbf{H}^{-1}\mathbf{X}\right|^{-1}
\end{aligned}
$$

Using the well-known formula for the determinant of block-matrix,

$$
\begin{aligned}
&= |\mathbf{H}|^{-1} \cdot \left|\mathbf{U}_{\mathrm{L}}^{\mathrm{T}}\mathbf{U}_{\mathrm{L}}\right|^{-1} \cdot \left|\mathbf{M}\mathbf{M}^{\mathrm{T}} - \mathbf{M}\mathbf{U}_{\mathrm{L}}\left(\mathbf{U}_{\mathrm{L}}^{\mathrm{T}}\mathbf{U}_{\mathrm{L}}\right)^{-1}\mathbf{U}_{\mathrm{L}}^{\mathrm{T}}\mathbf{M}^{\mathrm{T}}\right|^{-1} \cdot \left|\mathbf{X}^{\mathrm{T}}\mathbf{H}^{-1}\mathbf{X}\right|^{-1} \\
&= |\mathbf{H}|^{-1} \cdot \left|\mathbf{M}\mathbf{M}^{\mathrm{T}} - \mathbf{M}\mathbf{S}\mathbf{M}^{\mathrm{T}}\right|^{-1} \cdot \left|\mathbf{X}^{\mathrm{T}}\mathbf{H}^{-1}\mathbf{X}\right|^{-1} \\
&= |\mathbf{H}|^{-1} \cdot \left|\mathbf{M}\mathbf{X}\left(\mathbf{X}^{\mathrm{T}}\mathbf{X}\right)^{-1}\mathbf{X}^{\mathrm{T}}\mathbf{M}^{\mathrm{T}}\right|^{-1} \cdot \left|\mathbf{X}^{\mathrm{T}}\mathbf{H}^{-1}\mathbf{X}\right|^{-1} \\
&= |\mathbf{H}|^{-1} \cdot \left|\mathbf{X}^{\mathrm{T}}\mathbf{X}\right| \cdot \left|\mathbf{X}^{\mathrm{T}}\mathbf{H}^{-1}\mathbf{X}\right|^{-1}
\end{aligned}
$$

Therefore,

$$
\frac{1}{2}\log|\mathbf{Q}|_+ = -\frac{1}{2}\log|\mathbf{H}| + \frac{1}{2}\log\left|\mathbf{X}^{\mathrm{T}}\mathbf{X}\right| - \frac{1}{2}\log\left|\mathbf{X}^{\mathrm{T}}\mathbf{H}^{-1}\mathbf{X}\right|
$$

If we plus the both sides of the equation by $-(n-p)\log(\sigma_c^2)/2$, we get

$$
\frac{1}{2}\log\left|\mathbf{Q}/\sigma_c^2\right|_+ = -\frac{1}{2}\log|\mathbf{V}| + \frac{1}{2}\log\left|\mathbf{X}^{\mathrm{T}}\mathbf{X}\right| - \frac{1}{2}\log\left|\mathbf{X}^{\mathrm{T}}\mathbf{V}^{-1}\mathbf{X}\right|,
$$

which is also the frequently used form of the formula. □

Details about the material and sumulations

Details about the simulation of phenotype data

As described in the main text, the model as Eq 30 was used for the simulation of phenotypic values.

$$\mathbf{y} = \mathbf{X}_1\beta_1 + \mathbf{X}_2\beta_2 + \mathbf{X}_3\beta_3 + \mathbf{Z}\mathbf{u} + \mathbf{e}, \tag{30}$$

where $\mathbf{y}$ is the vector of simulated phenotypic values of 414 accessions, $\mathbf{X}_1$, $\mathbf{X}_2$ and $\mathbf{X}_3$ correspond to three quantitative trait nucleotides (QTNs) scored as -1, 0 or 1 (hereinafter, referred to as "QTN1", "QTN2" and "QTN3" respectively), β_1, β_2 and β_3 are scalars representing the effects of the three QTNs, $\mathbf{u}$ is the vector of polygenetic effects, and $\mathbf{e}$ is the vector of the residuals. Here, we will explain the details about the last two terms.

The polygenetic effects $\mathbf{u}$ in Eq 31 were sampled from the multivariate normal distribution whose variance-covariance matrix was the additive genetic relationship matrix $\mathbf{A}$, and were normalized so that their variance was equal to that of three QTN effects.

$$\mathbf{u} \sim \mathrm{MVN}(\mathbf{0}, \mathbf{G}) \tag{31}$$

$\mathbf{G} = \mathbf{A}\sigma^2_{\mathrm{A}}$ is the genetic covariance matrix, and the additive genetic variance σ^2_{A} is automatically determined from the relationship with the heritability. In this study, the additive numerator relationship matrix was estimated based on the marker genotype of 112,630 SNPs using the "A.mat" function of the R package "rrBLUP" version 4.6 [16].

The residual $\mathbf{e}$ in Eq 32 was sampled identically and independently from a normal distribution, and then was normalized so that the narrow-sense heritability was equal to 0.6.

$$\mathbf{e} \sim \mathrm{MVN}(\mathbf{0}, \mathbf{I}\sigma^2_{\mathrm{e}}), \tag{32}$$

where $\mathbf{I}$ is an identity matrix, and σ^2_{e} is the residual variance determined so that the heritability is equal to 0.6.

Details about the evaluation of RAINBOW

Details of four methods

Here, we will explain the details of four methods for the evaluation of the RAINBOW performance.

Single-SNP GWAS The single-SNP GWAS model was similar to Eq 2, but this method regarded each SNP as a fixed effect and tested the significance of each SNP effect one by one [17]. In this study, we used the method called "P3D", which first estimated σ^2_{c} and σ^2_{e} by solving the mixed model without SNP effects (Eq 2) by REML, and then tested each marker using the information of the estimated variance $\hat{\sigma}^2_{\mathrm{c}}$ and $\hat{\sigma}^2_{\mathrm{e}}$ [6, 18, 19]. We used the "GWAS" function of the R package "rrBLUP" version 4.6 [16].

HGF method HGF tests the significance of each haplotype block effect. In this method, all accessions were divided into k_{r_i} groups based on the haplotypes. The haplotype groups were then represented by dummy variables. The significance of the haplotype effect was tested by regarding a matrix of dummy variables as fixed effects [20]. In this study, the k-medoids method (the "pam" function of the R package "cluster" version 2.0.6) [21] or UPGMA (unweighted pair group method with arithmetic mean, "hclust" and "cutree" function of the R package "stats") [22] were employed to group haplotypes. We set the number of groups k_{r_i} as 2,3 or 4. Therefore, we applied 6 different HGF models in total. To apply HGF models, we modified the R package "rrBLUP" version 4.6 to enable the use of a matrix of dummy variables as fixed effects [16].

SKAT The SKAT is a SNP-set GWAS method, and is widely used in human genetics [23]. This method was invented to detect rare alleles, but the SKAT model does not include the random effects term that accounts for family relatedness, which may cause false positives for GWAS in materials with a strong population structure or family relatedness. In this study, we used the "SKAT" function of the R package "SKAT" version 1.3.2.1 and performed SNP-set GWAS by regarding each haplotype block as each SNP-set.

RAINBOW The methods for RAINBOW were described in the main text. In this study, we performed haplotype-based GWAS for RAINBOW by regarding each haplotype block as each SNP-set. We used the linear kernel of each haplotype block for the Gram matrix $\mathbf{K}_{\mathrm{r}_i}$.

References

[1] Patterson HD, Thompson R. Recovery of inter-block information when block sizes are unequal. Biometrika. 1971;58(3):545 –554.

[2] Harville DA. Bayesian Inference for Variance Components Using Only Error Contrasts. Biometrika. 1974;61(2):383–385.

[3] Sorensen D, Gianola D. Likelihood, Bayesian, and MCMC Methods in Quantitative Genetics. Heidelberg, New York: Springer; 2002.

[4] Crobeil RR, Searle SR. Restricted Maximum Likelihood (REML) Estimation of Variance Components in the Mixed Model. Technometrics. 1976;18(1):31–38.

[5] Welham SJ, Thompson R. Likelihood ratio tests for fixed model terms using residual maximum likelihood. J R Stat Soc Ser B Stat Methodol. 1997;59(3):701–714.

[6] Kang HM, Zaitlen NA, Wade CM, Kirby A, Heckerman D, Daly MJ, et al. Efficient Control of Population Structure in Model Organism Association Mapping. Genetics. 2008;178(3):1709–1723.

[7] Neyman J, Pearson ES. On the Use and Interpretation of Certain Test Criteria for Purposes of Statistical Inference. Biometrika. 1928;20A(1-2):175–240.

[8] Wilks SS. The Large-Sample Distribution of the Likelihood Ratio for Testing Composite Hypotheses. Ann Math Stat. 1938;9(1):60–62.

[9] Listgarten J, Lippert C, Kang EY, Xiang J, Kadie CM, Heckerman D. A powerful and efficient set test for genetic markers that handles confounders. Bioinformatics. 2013;29(12):1526–1533.

[10] Lippert C, Xiang J, Horta D, Widmer C, Kadie C, Heckerman D, et al. Greater power and computational efficiency for kernel-based association testing of sets of genetic variants. Bioinformatics. 2014;30(22):3206–3214.

[11] Byrd R, Lu P, Nocedal J, Zhu C. A Limited Memory Algorithm for Bound Constrained Optimization. SIAM Journal on Scientific Computing. 1995;16(5):1190–1208.

[12] Zhou X, Stephens M. Genome-wide efficient mixed-model analysis for association studies. Nat Genet. 2012;44(7):821–824.

[13] Endelman JB, Jannink JL. Shrinkage Estimation of the Realized Relationship Matrix. G3 (Bethesda). 2012;2(11):1405–1413.

[14] Su G, Christensen OF, Ostersen T, Henryon M, Lund MS. Estimating Additive and Non-Additive Genetic Variances and Predicting Genetic Merits Using Genome-Wide Dense Single Nucleotide Polymorphism Markers. PLoS One. 2012;7(9):1–7.

[15] Henderson CR. Best Linear Unbiased Prediction of Nonadditive Genetic Merits in Noninbred Populations. J Anim Sci. 1985;60(1):111–117.

[16] Endelman JB. Ridge Regression and Other Kernels for Genomic Selection with R Package rrBLUP. Plant Genome J. 2011;4(3):250.

[17] Yu J, Pressoir G, Briggs WH, Vroh Bi I, Yamasaki M, Doebley JF, et al. A unified mixed-model method for association mapping that accounts for multiple levels of relatedness. Nat Genet. 2006;38(2):203–8.

[18] Kang HM, Sul JH, Service SK, Zaitlen NA, Kong SY, Freimer NB, et al. Variance component model to account for sample structure in genome-wide association studies. Nat Genet. 2010;42(4):348–354.

[19] Zhang Z, Ersoz E, Lai CQ, Todhunter RJ, Tiwari HK, Gore MA, et al. Mixed linear model approach adapted for genome-wide association studies. Nat Genet. 2010;42(4):355–360.

[20] Yano K, Yamamoto E, Aya K, Takeuchi H, Lo PC, Hu L, et al. Genome-wide association study using whole-genome sequencing rapidly identifies new genes influencing agronomic traits in rice. Nat Genet. 2016;48(8):927–934.

[21] Maechler M, Rousseeuw P, Struyf A, Hubert M, Hornik K. cluster: Cluster Analysis Basics and Extensions; 2018.

[22] R Core Team. R: A Language and Environment for Statistical Computing; 2019. Available from: https://www.R-project.org/.

[23] Wu MC, Lee S, Cai T, Li Y, Boehnke M, Lin X. Rare-variant association testing for sequencing data with the sequence kernel association test. Am J Hum Genet. 2011;89(1):82–93.
