# Supplementary material for: RAINBOW: Haplotype-based genome-wide association study using a novel SNP-set method
Source: PLoS Comput Biol. 2020 Feb 14;16(2):e1007663. doi: 10.1371/journal.pcbi.1007663 (PMC7046296; doi:10.1371/journal.pcbi.1007663)
Supplement: S1 Fig — (PDF) [file pcbi.1007663.s004.pdf]

✤ Simulation of phenotypic values (**100** iterations)

$$\mathbf{y} = \mathbf{X}_1\beta_1 + \mathbf{X}_2\beta_2 + \mathbf{X}_3\beta_3 + \mathbf{Zu} + \mathbf{e}, \text{(Eq. 1)}$$

$\mathbf{y}$: phenotypic values	$\mathbf{X}_1\beta_1$, $\mathbf{X}_2\beta_2$, $\mathbf{X}_3\beta_3$: fixed effects (marker effects)
$\mathbf{Zu}$: random effects (polygene effects)	$\mathbf{e}$: residuals

- $\mathbf{X}_1$ and $\mathbf{X}_2$ belong to the same haplotype block.

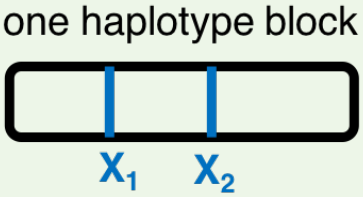

✤ We assumed 2 scenarios.

① **Coupling** : cor($\mathbf{X}_1$, $\mathbf{X}_2$) > 0 -> $\beta_1 = \beta_2$, cor($\mathbf{X}_1$, $\mathbf{X}_2$) < 0 -> $\beta_1 = -\beta_2$

② **Repulsion** : cor($\mathbf{X}_1$, $\mathbf{X}_2$) > 0 -> $\beta_1 = -\beta_2$, cor($\mathbf{X}_1$, $\mathbf{X}_2$) < 0 -> $\beta_1 = \beta_2$

✤ Perform GWAS with 4 methods.

① Our proposed method, **RAINBOW** (R package RAINBOWR)

② The **single-SNP** method (R package rrBLUP)

③ **Haplotype-based** method introduced by Yano *et al.*, 2016

④ **SNP-set** method, SKAT (R package SKAT)

✤ Evaluate the results with the following summary statistics

① $-\log_{10}(p)$ and $-\log_{10}(p_a)$, correspond to the **detection power**

② **Recall**, **Precision**, and **F-measure**

③ **AUC** (area under the curve) for **regions around causals**
