# Supplementary material for: RAINBOW: Haplotype-based genome-wide association study using a novel SNP-set method
Source: PLoS Comput Biol. 2020 Feb 14;16(2):e1007663. doi: 10.1371/journal.pcbi.1007663 (PMC7046296; doi:10.1371/journal.pcbi.1007663)
Supplement: S2 Fig — How to view this figure (including legends and abbreviations) is the same as that of Fig 1. (PDF) [file pcbi.1007663.s005.pdf]

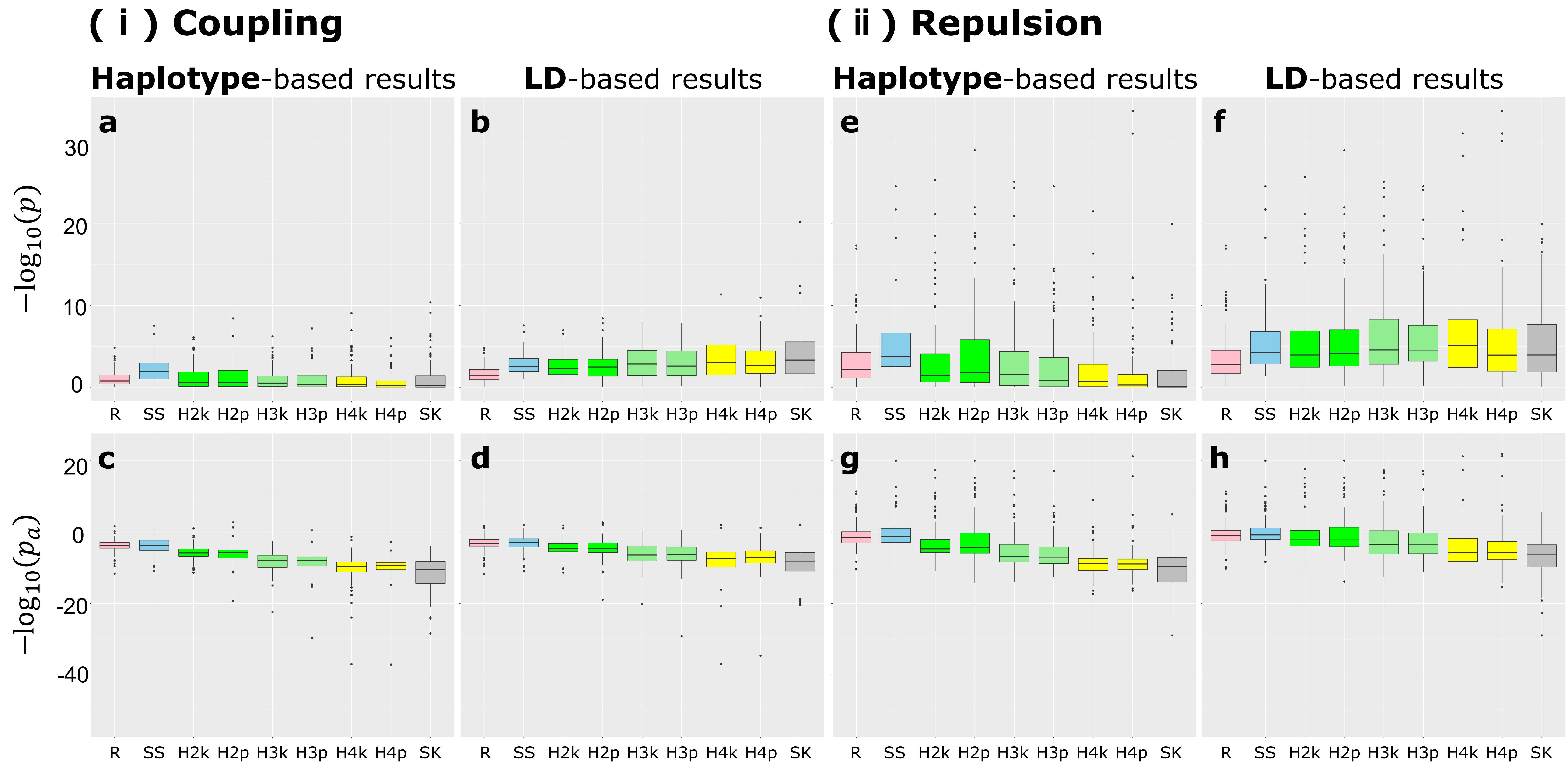

(i) Coupling
(ii) Repulsion
Haplotype-based results
LD-based results
Haplotype-based results
LD-based results
a
b
c
d
e
f
g
h
$-\log_{10}(p)$
$-\log_{10}(p_a)$
30
20
10
0
-20
-40
R
SS
H2k
H2p
H3k
H3p
H4k
H4p
SK
