# Supplementary figures and images for: RAINBOW: Haplotype-based genome-wide association study using a novel SNP-set method
Source: PLoS Comput Biol. 2020 Feb 14;16(2):e1007663. doi: 10.1371/journal.pcbi.1007663 (PMC7046296; doi:10.1371/journal.pcbi.1007663)

**(a) Coupling**

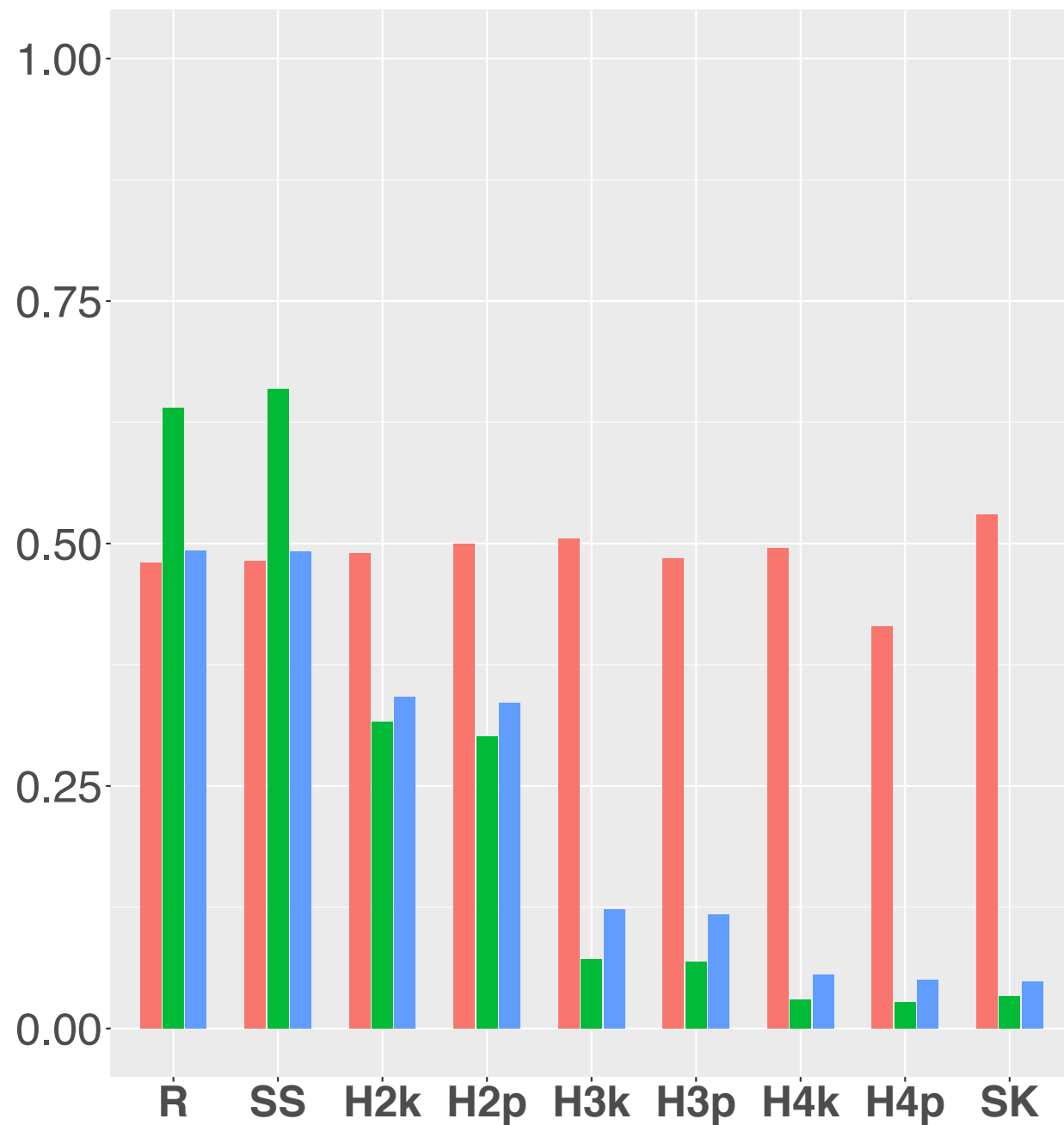

**(b) Repulsion**

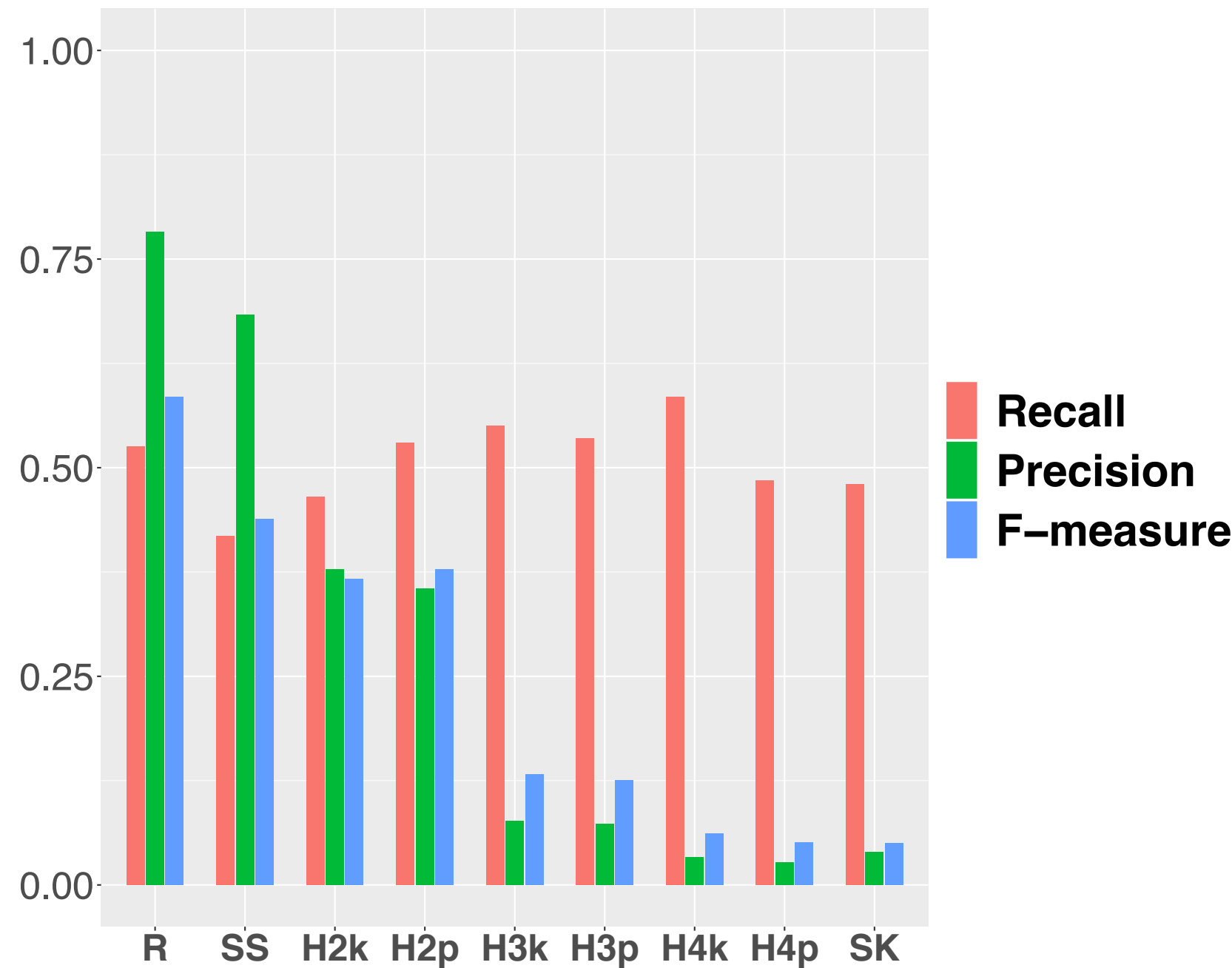

Supplement: S3 Fig — How to view this figure (including legends and abbreviations) is the same as that of Fig 2. (PDF) [file pcbi.1007663.s006.pdf]

**( i ) Coupling**

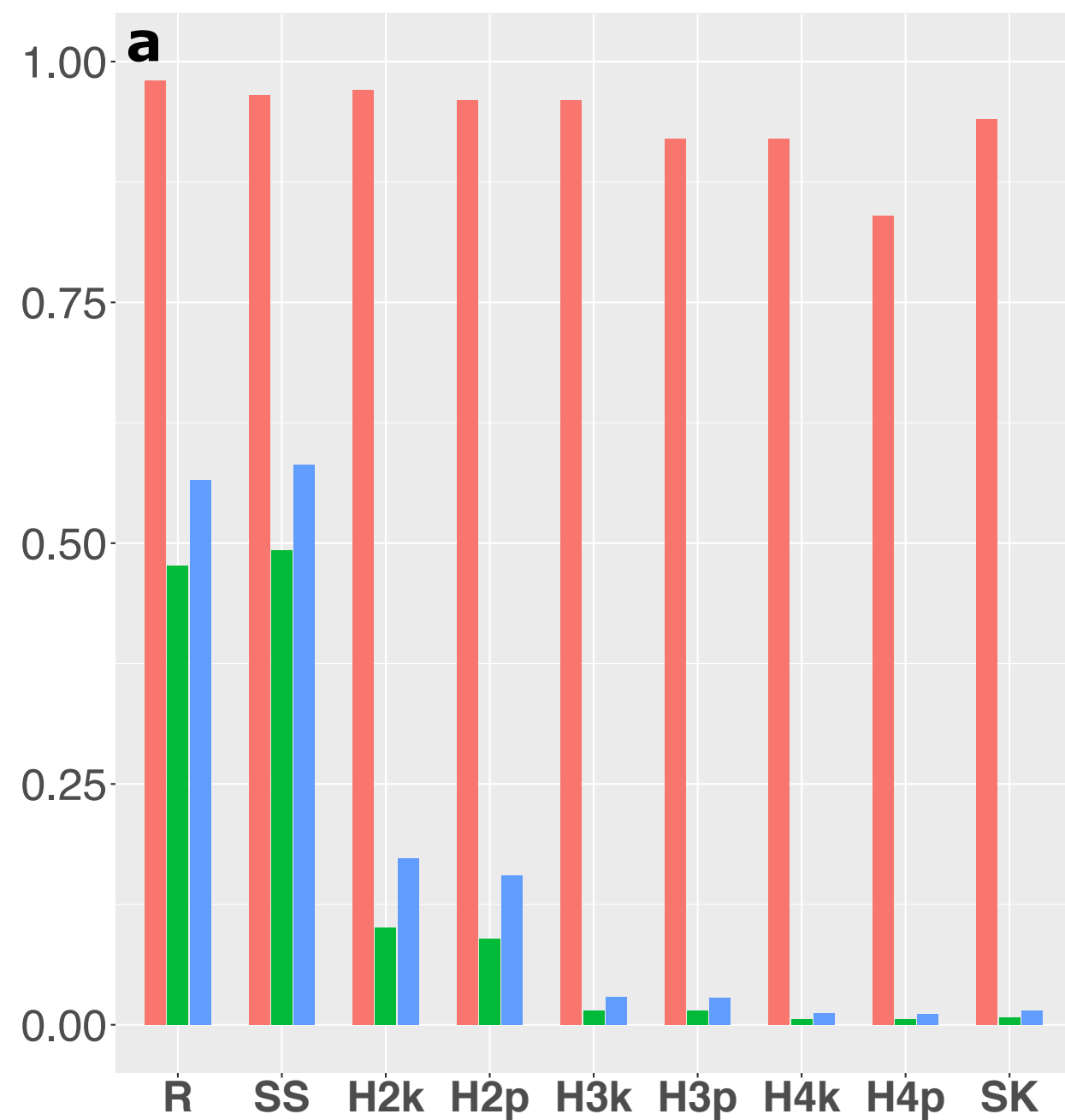

**( ii ) Repulsion**

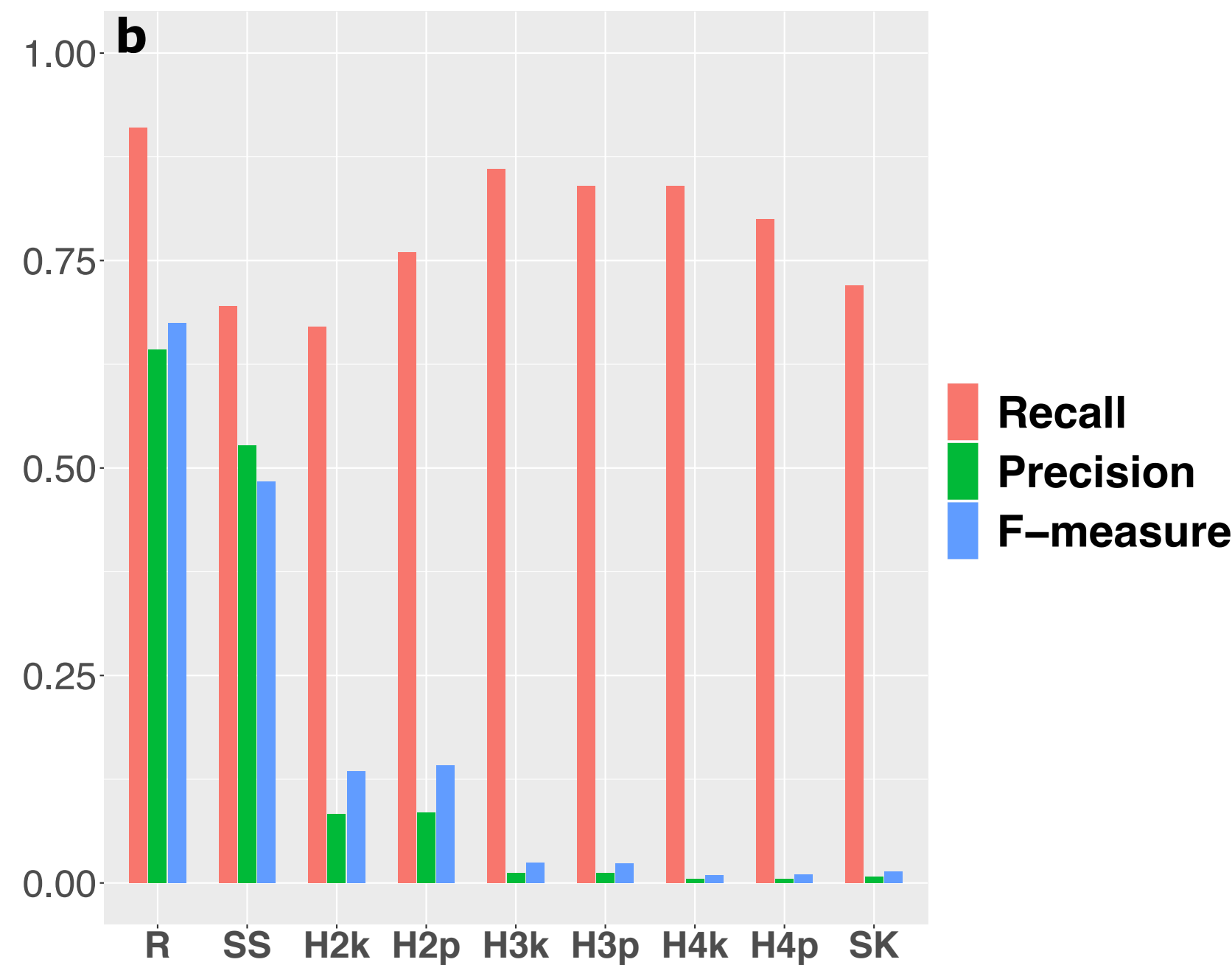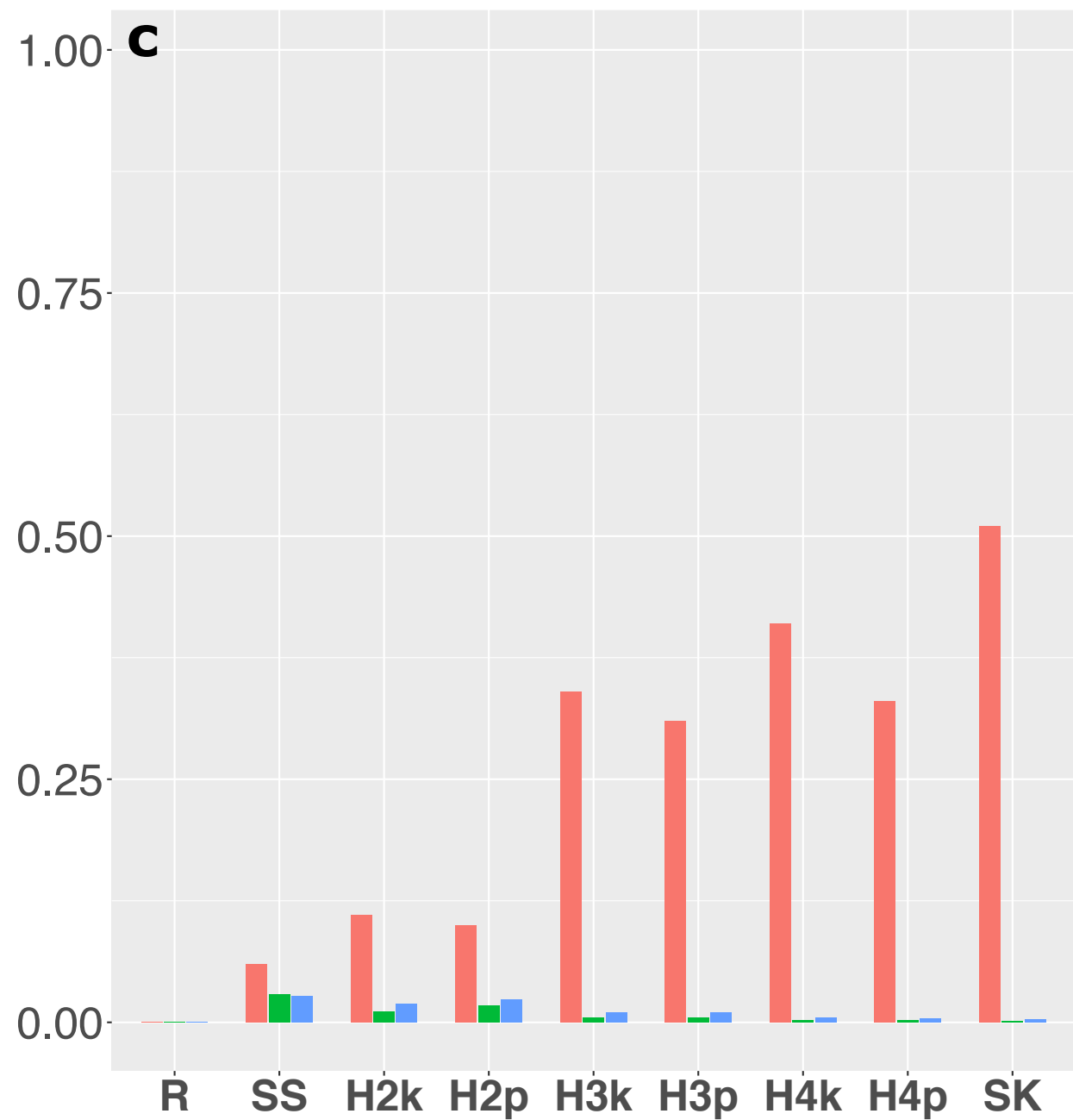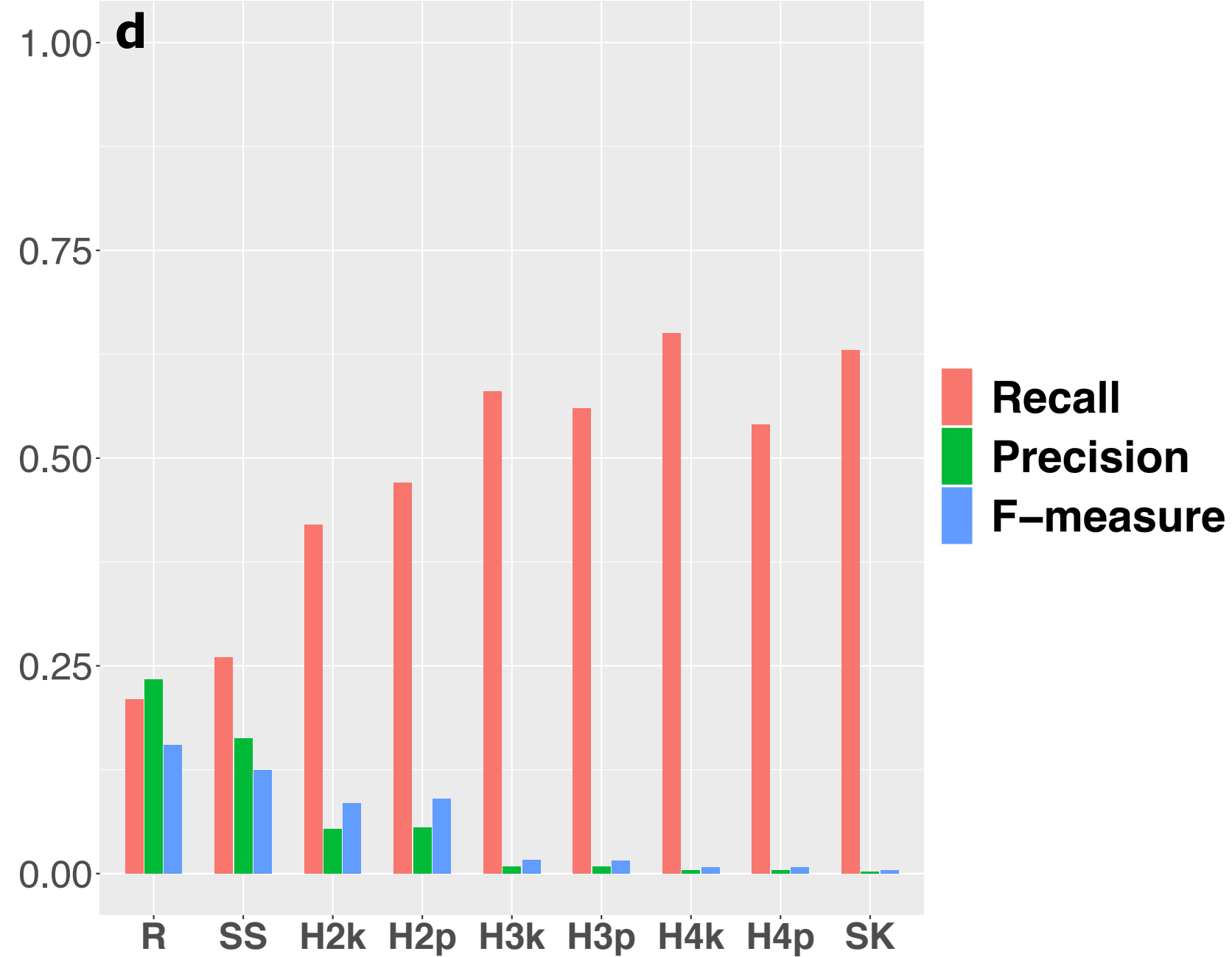

Supplement: S4 Fig — How to view this figure (including legends and abbreviations) is the same as that of Fig 2. (PDF) [file pcbi.1007663.s007.pdf]

**( i ) Coupling**

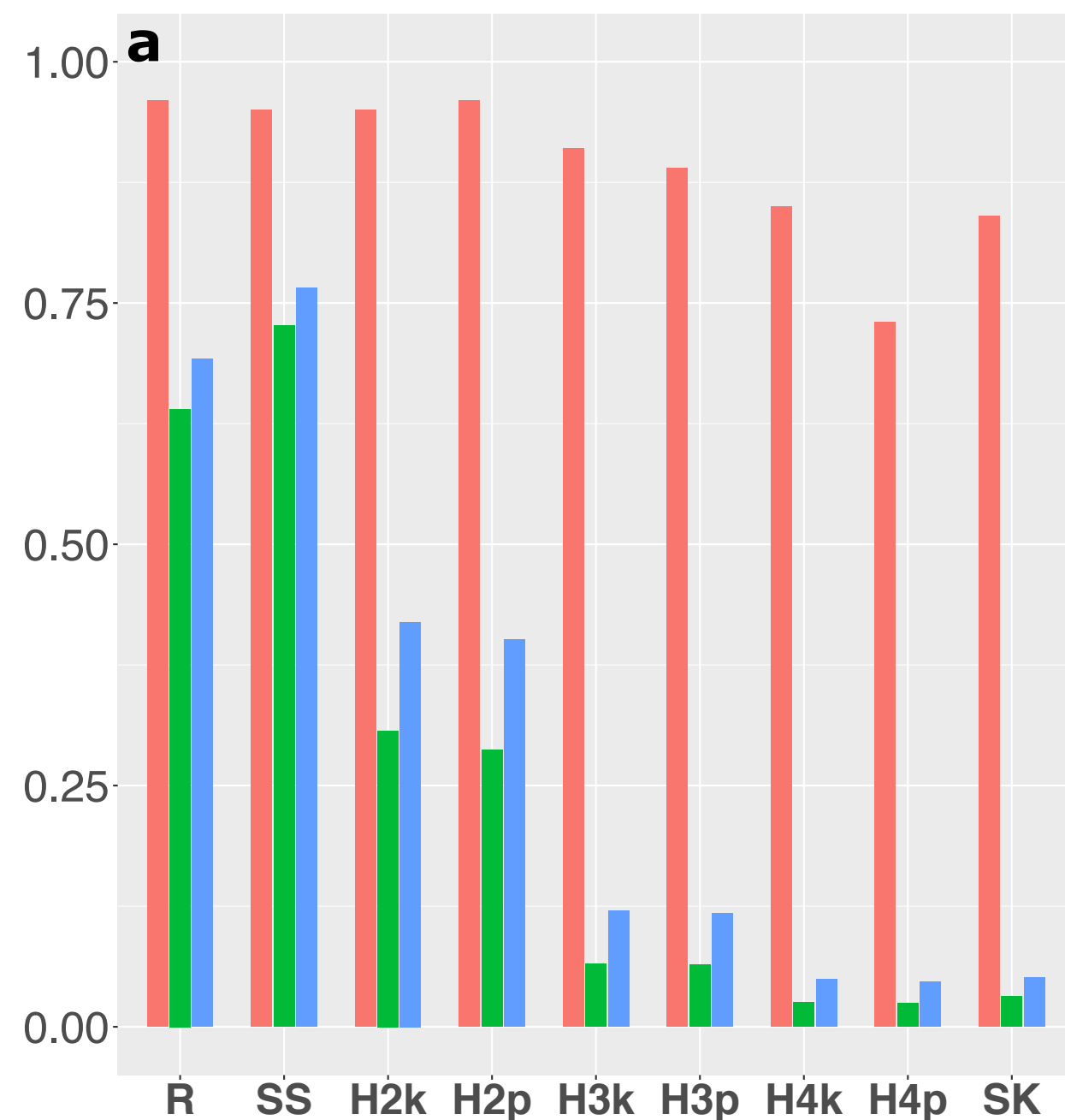

**( ii ) Repulsion**

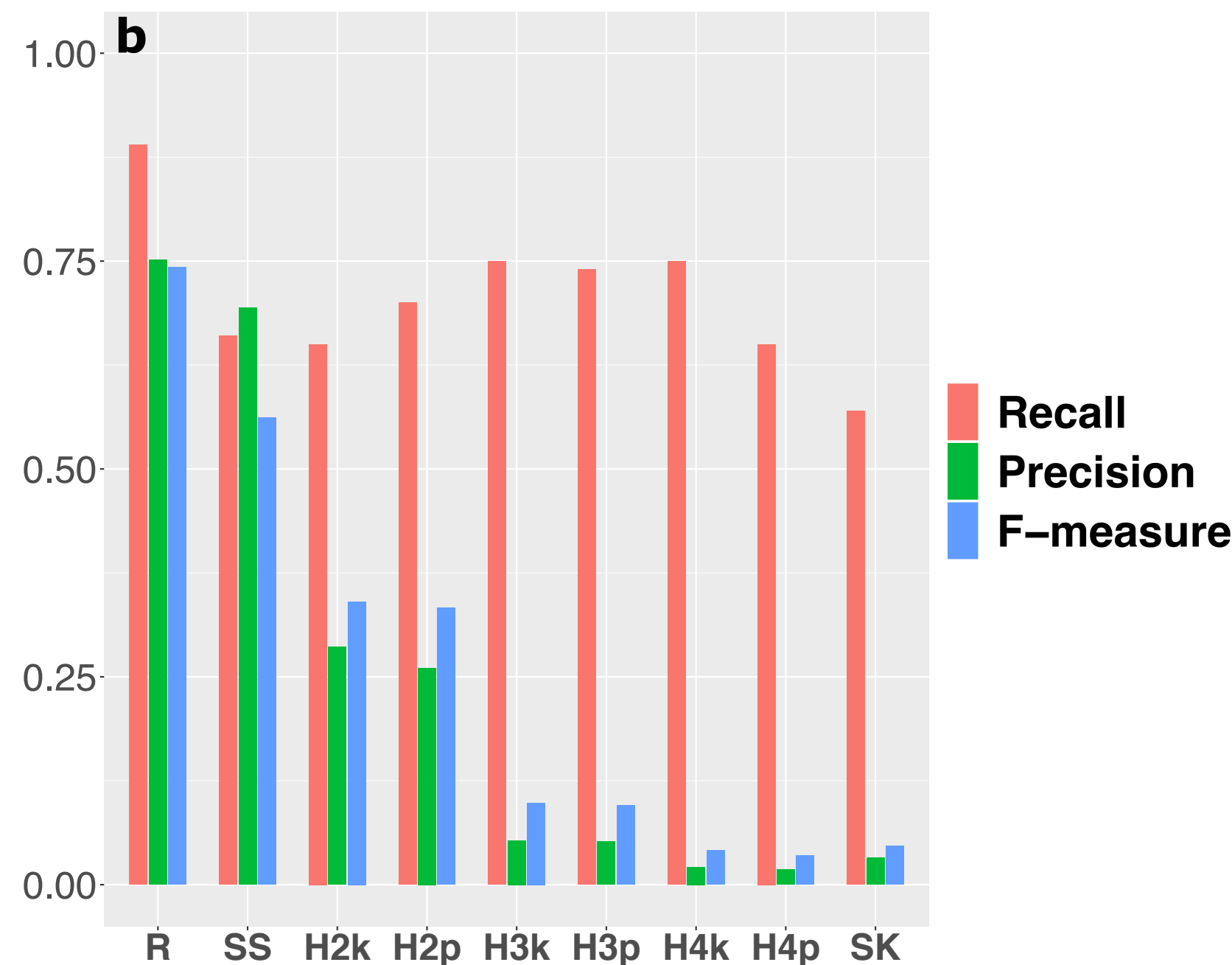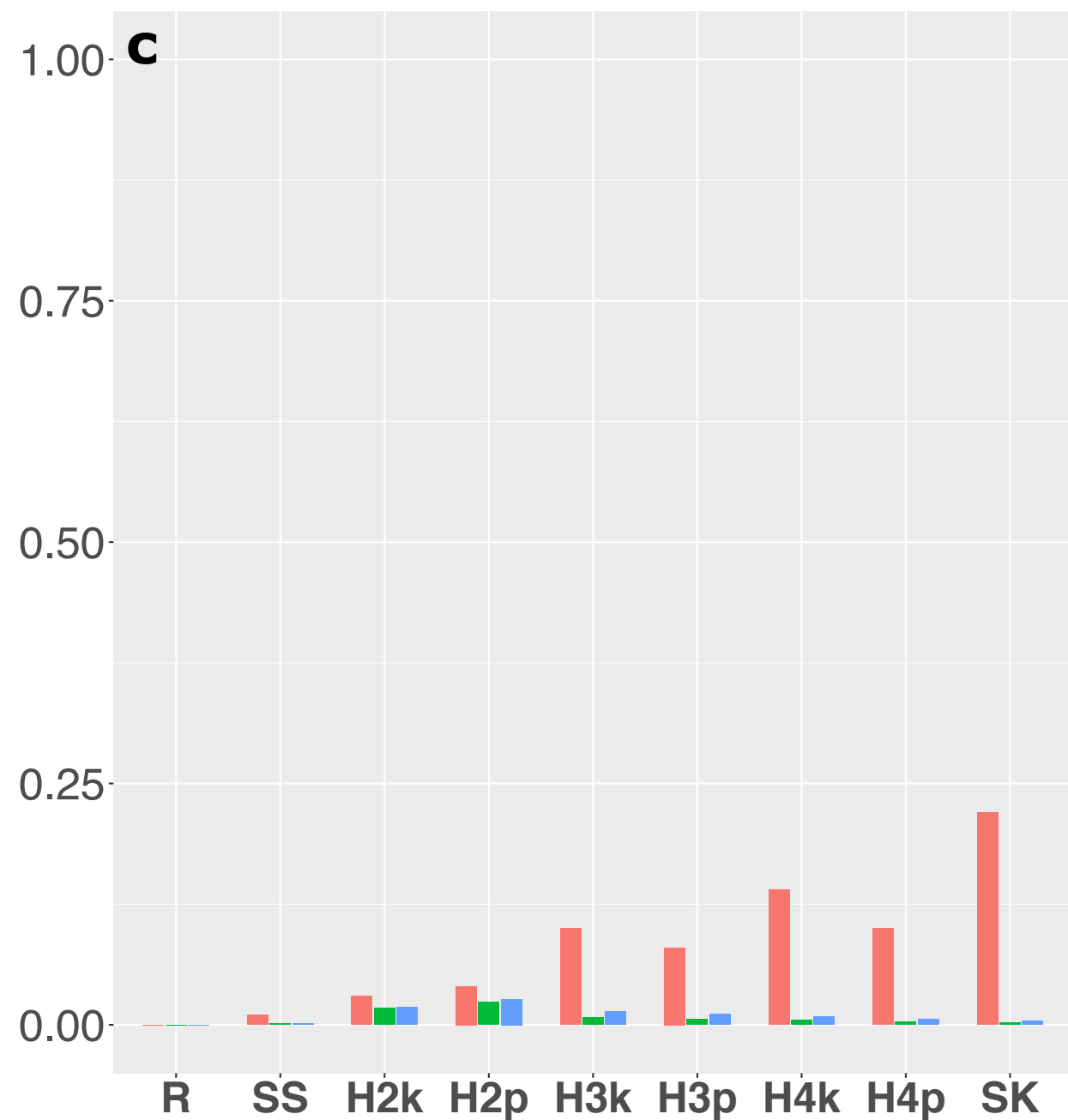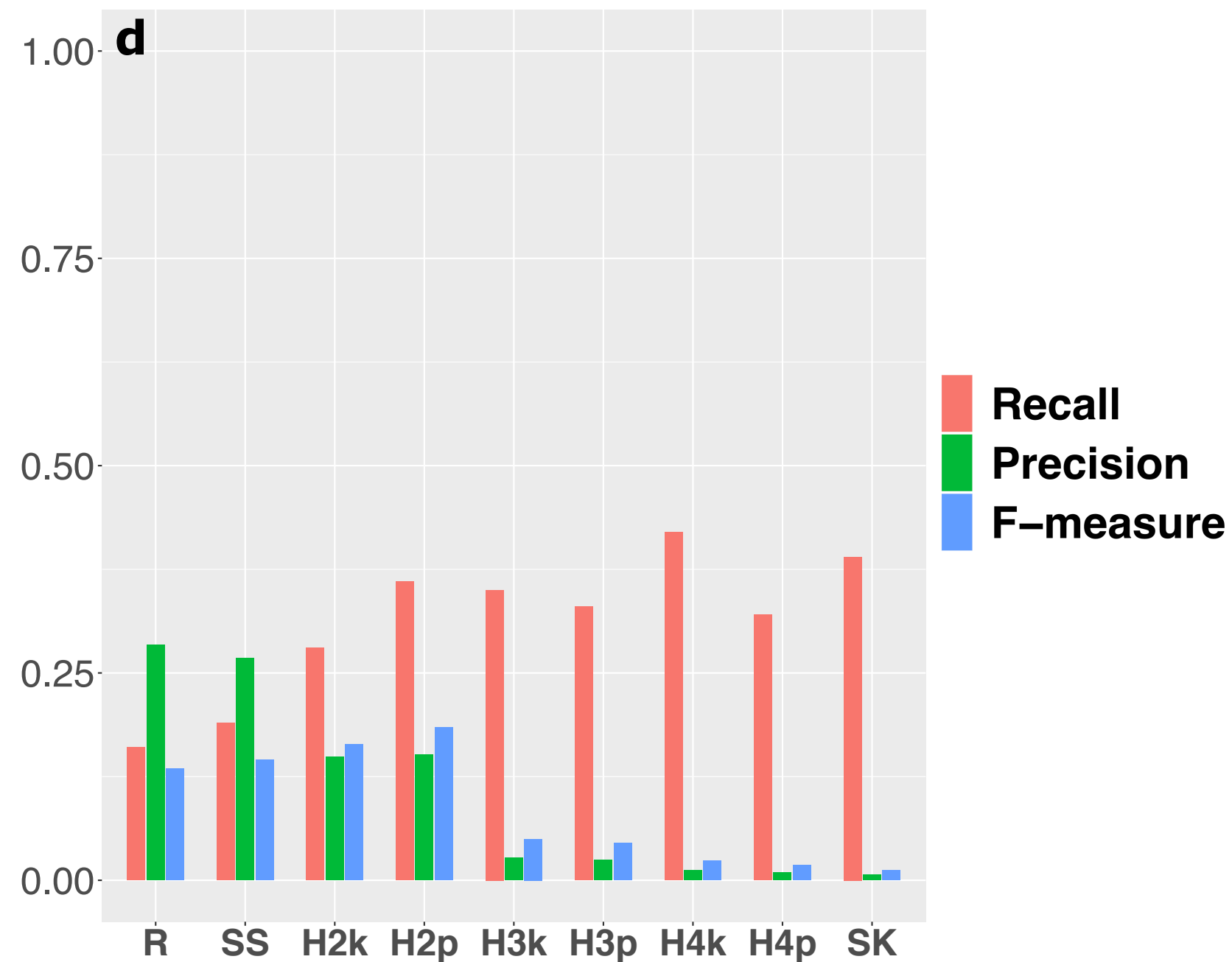

Supplement: S5 Fig — How to view this figure (including legends and abbreviations) is the same as that of Fig 2. (PDF) [file pcbi.1007663.s008.pdf]
