# Supplementary material for: RAINBOW: Haplotype-based genome-wide association study using a novel SNP-set method
Source: PLoS Comput Biol. 2020 Feb 14;16(2):e1007663. doi: 10.1371/journal.pcbi.1007663 (PMC7046296; doi:10.1371/journal.pcbi.1007663)

Iteration 40

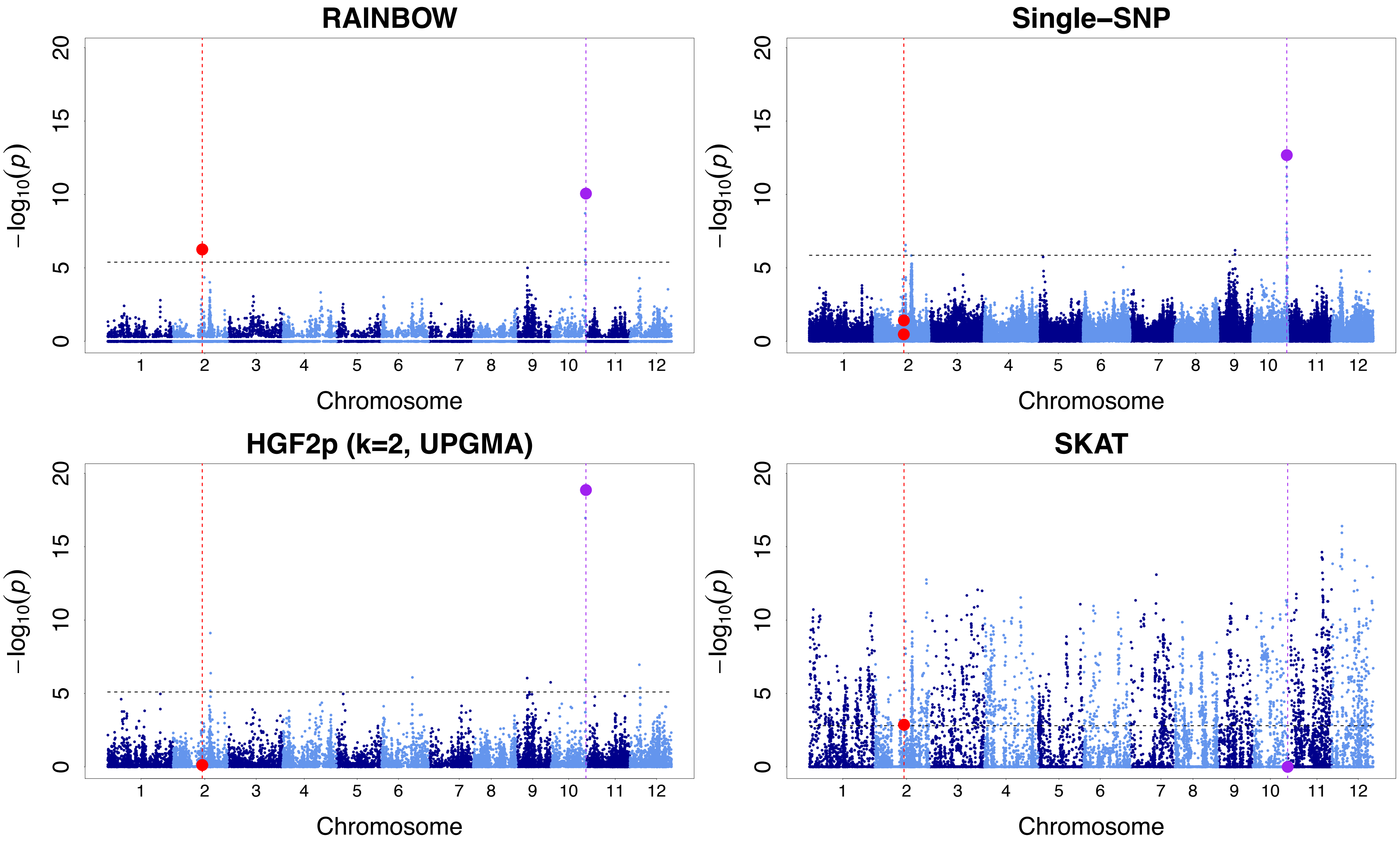

Iteration 43

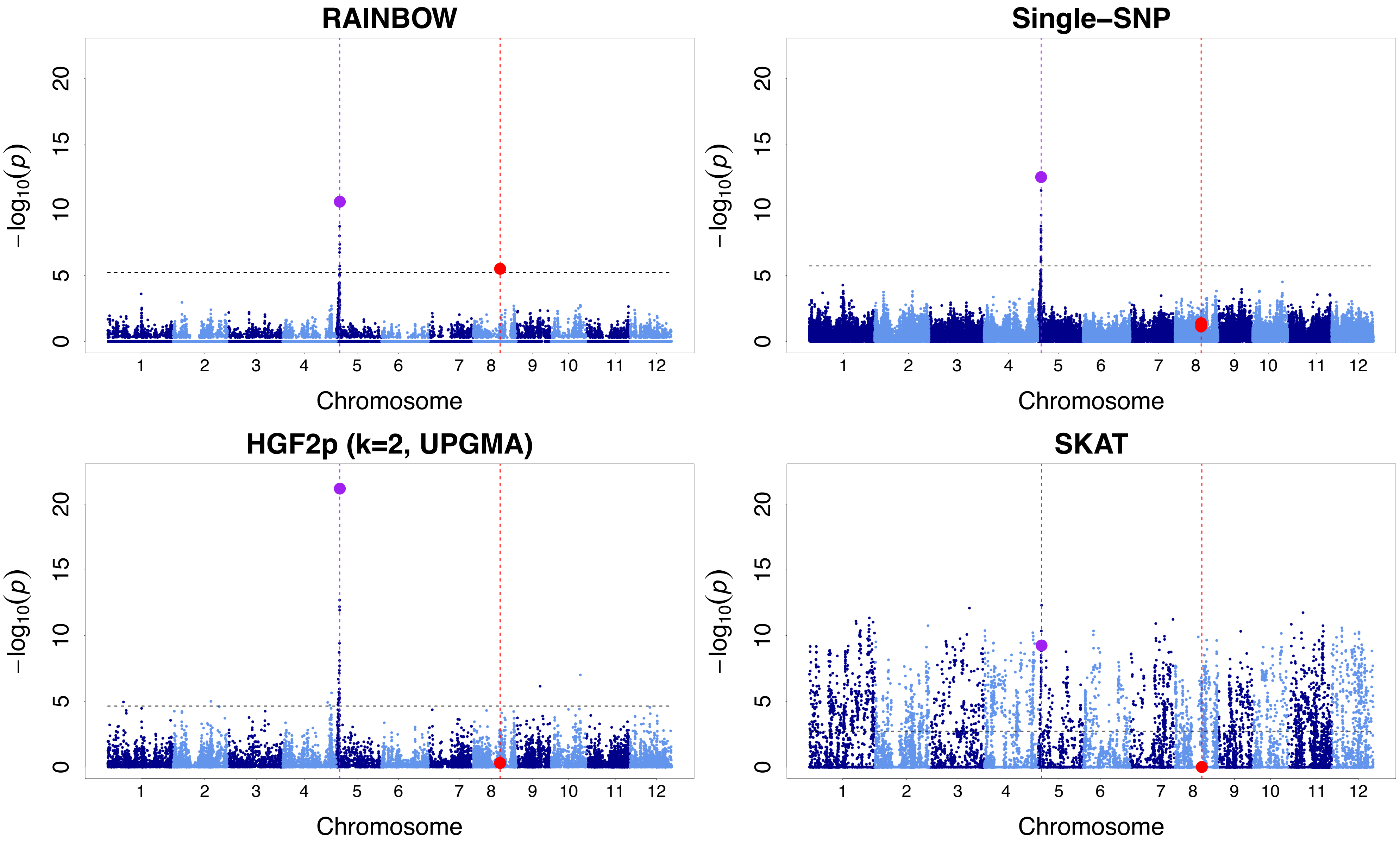

Iteration 64

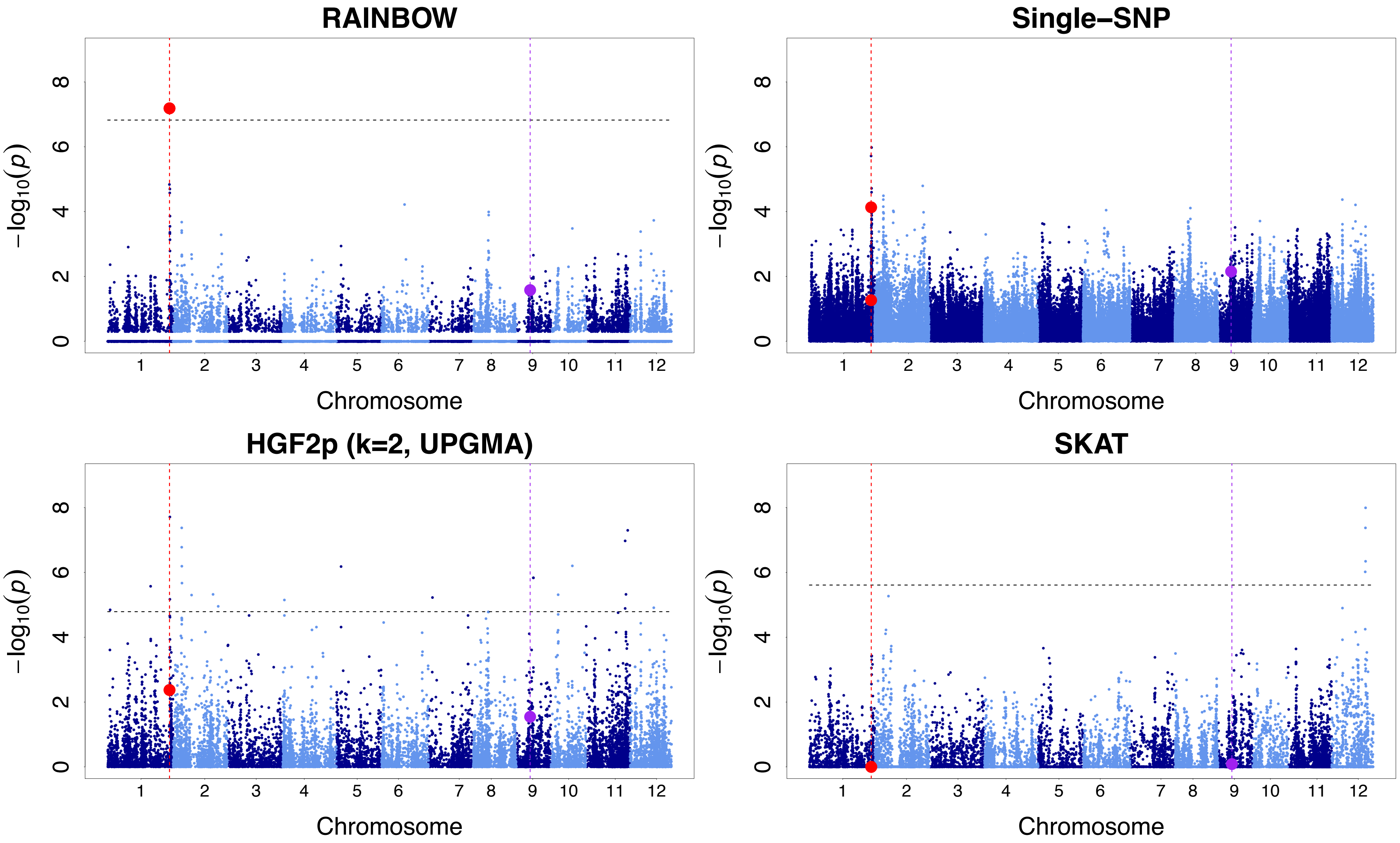

Iteration 69

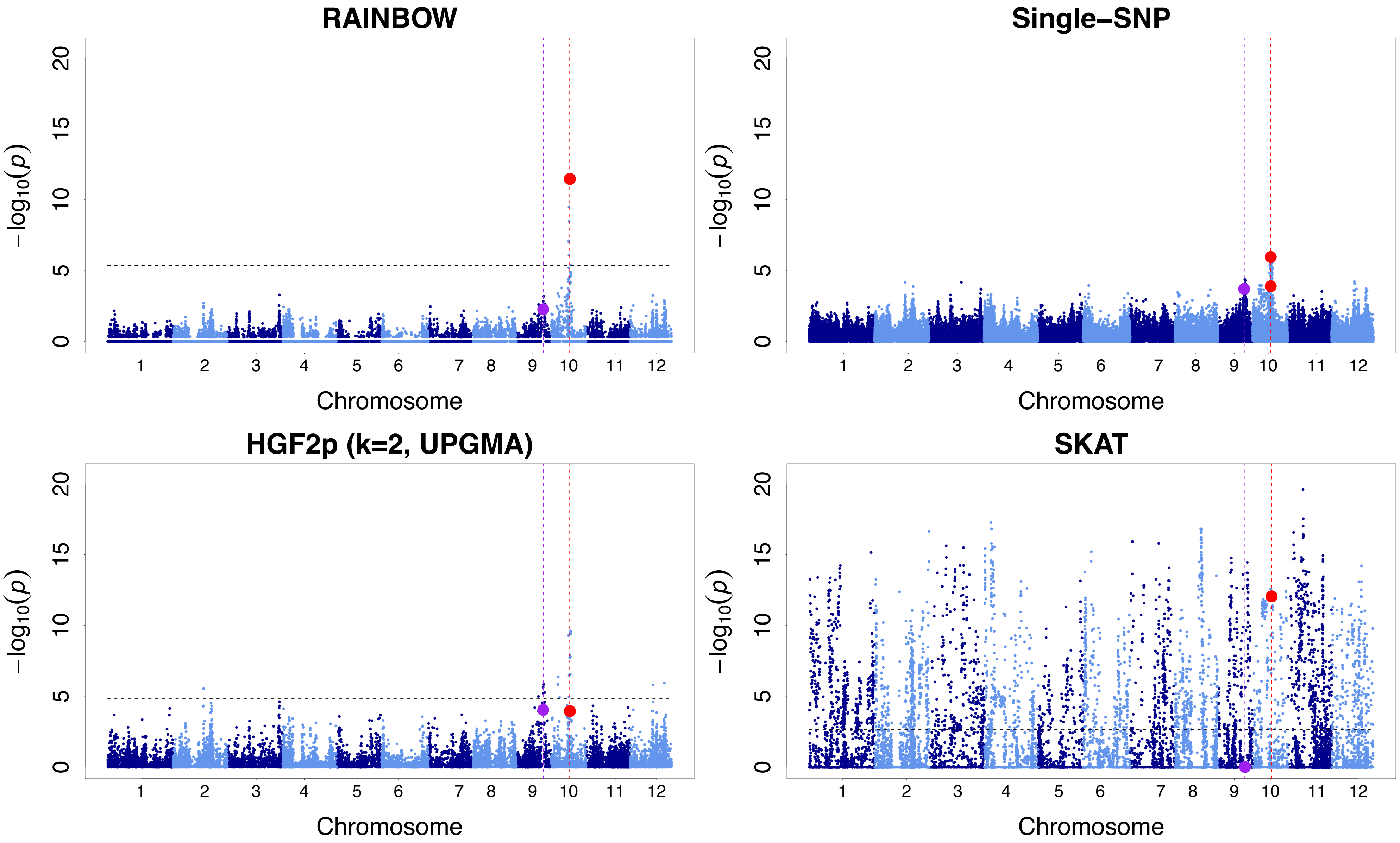

Iteration 81

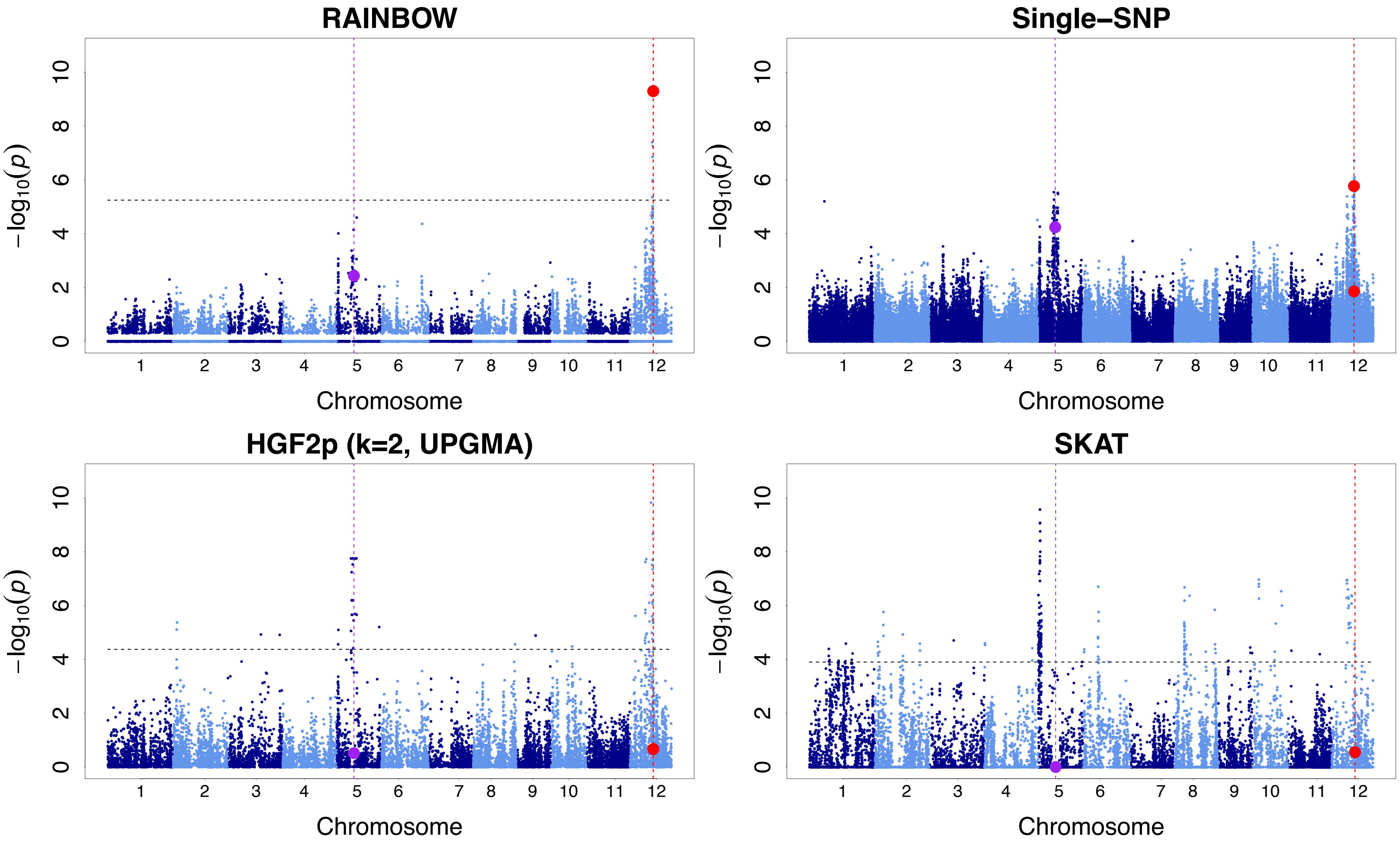

Iteration 85

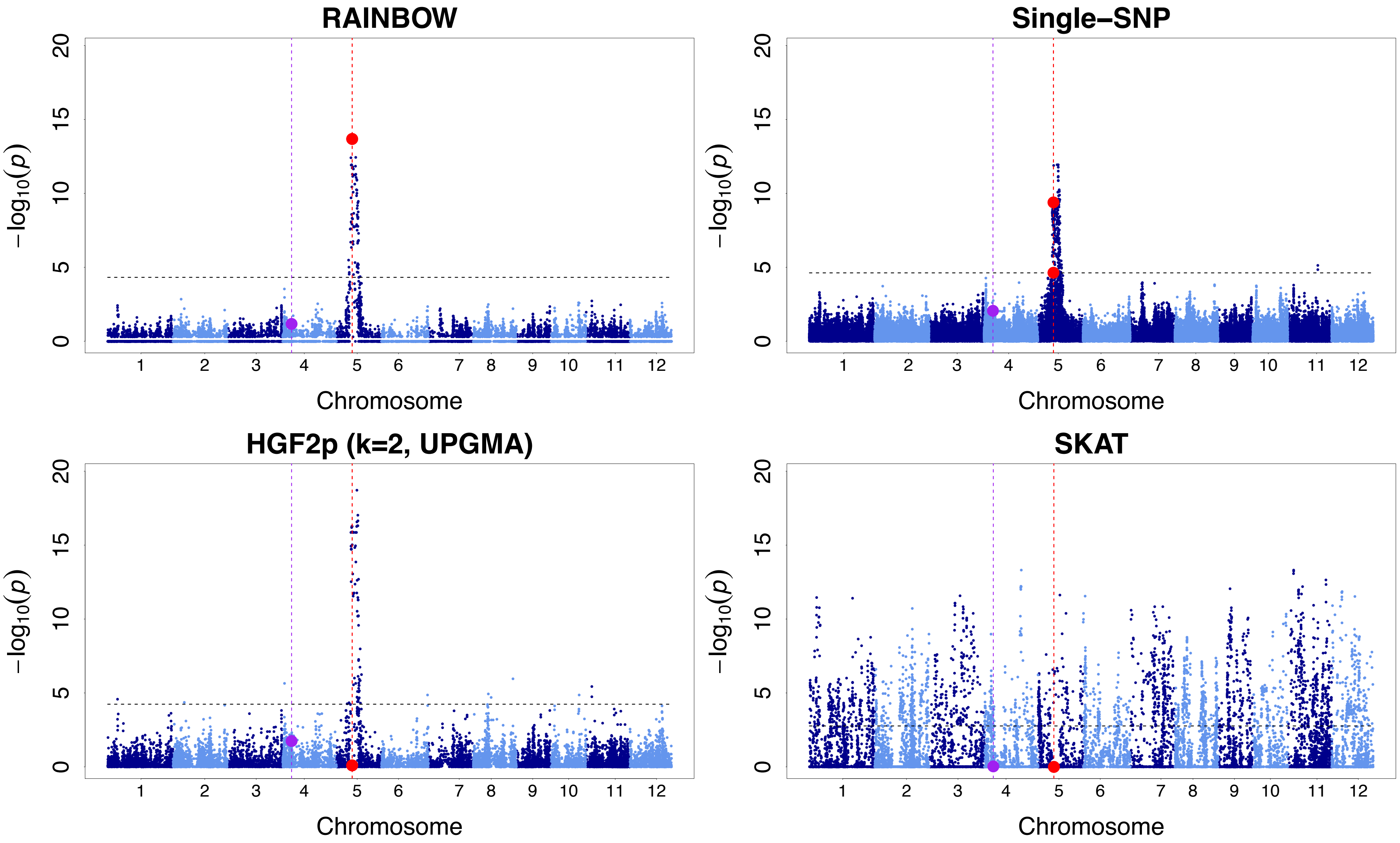

Supplement: S6 Fig — How to view this figure (including legends and abbreviations) is the same as that of Fig 4. (PDF) [file pcbi.1007663.s009.pdf]
