# Supplementary material for: RAINBOW: Haplotype-based genome-wide association study using a novel SNP-set method
Source: PLoS Comput Biol. 2020 Feb 14;16(2):e1007663. doi: 10.1371/journal.pcbi.1007663 (PMC7046296; doi:10.1371/journal.pcbi.1007663)
Supplement: Attachment — Submitted filename: Review.pdf [file pcbi.1007663.s010.pdf]

PCOMPBIOL-D-19-01767

RAINBOW: Haplotype-based genome-wide association study using a novel SNP-set method

Authors introduce a method, implemented as an R-package RAINBOW, to detect a single or set of potential causal SNPs in GWAS. To summarise briefly, RAINBOW method employs a linear mixed model approach; uses a likelihood ratio test to test for a single/set of SNPs fitted as a random effect where the corresponding variance-covariance matrix is computed to be proportional to the Gram matrix from the marker genotype data; takes into account family relatedness by inclusion of another random effect with corresponding variance-covariance matrix given to be proportional to the numerator relationship matrix $\mathbf{A}$.

To fit this model in this practice, RAINBOW converts the model to a single random-effect model by using a weighted average of the variance-covariance matrices of the $\mathbf{Z}_c\boldsymbol{u}_c$ (all markers) and $\mathbf{Z}_{r_i}\boldsymbol{u}_{r_i}$ (markers tested) to be proportional to the variance-covariance of the random effect. This single random-effect model is fitted in EMMA/GEMMA. This process is repeated to find optimal weights using L-BFGS optimization of full/restricted log likelihood.

Their method to fit their model, which is rather ad-hoc, I suspect arise from the software restriction of EMMA/GEMMA that can only fit a single random effect model. The aspect of this fitting process that I question is *how good are the resulting estimates compared to say if the model parameters were estimated directly via REML?* Some existing software such as SAS and asreml are able to estimate variance components directly via REML even when there are multiple random effects. These are of course commercial software and likely authors would like to have RAINBOW without those restrictions but I think a comparison of variance component estimates that authors obtain to REML estimates are warranted.

General comment:

- ρ_{12} for Eqns (16) and (17) does not seem to be defined. I assumed to be Pearson's correlation coefficient between $\mathbf{X}_1$ and $\mathbf{X}_2$ but could the authors clarify?
- P4. L82 & L89 & L95. σ_c^2 and $\sigma_{r_i}^2$ are *not* estimated by solving the mixed effect model Eq (1). It is important to distinguish between the *model* and the *fitting process* of the model. The fitting process estimates the variance parameters

usually using REML, but not always the case as is the authors' case described in "Estimation of variance components" section. The authors could just refer to "Estimation of variance components" section rather than referring that it is solved by model Eq (1).

- Any reference to back up the statement "the score test is not necessarily the best method for testing the random effects in the mixed effects model" in L41?

Citations

- Please cite software used. E.g. cite R; and cite ggplot2 for creating the figures. Citations for R-packages are easily obtained in R by using say `citation("ggplot2")` for `gglot2` R package.

- Please cite relevant statistical papers. E.g. REML attributed to Patterson & Thompson (1971); testing of variance components at boundary -> the asymptotic distribution discussed in Self and Liang (1987) with further discussion in Stram and Lee (1994) (latter may be more relevant as it pertains directly to LR tests. Score test, ML and so on also missing citations.

Minor comments:

Abstract:

- "The results indicated that the proposed method was able to control false positives than the others." -> control false positives *better* than other methods.

- "The proposed method was also excellent at detecting causal variants without relying on the linkage disequilibrium if causal variants were genotyped in the dataset. Moreover, the proposed method showed greater power than the other methods, i.e., it was able to detect causal variants that were not detected by the others, primarily when the causal variants were located very close to each other, and the directions of their effects were opposite. The proposed method, RAINBOW, is exceptionally superior in controlling false positives, detecting causal variants, and detecting nearby causal variants with opposite effects. By using the SNP-set approach as the proposed method, we expect that detecting not only rare variants but also genes with complex mechanisms, such as genes with multiple causal variants, can be realized." -> Rather than repeating "the proposed method" so many times, I suggest you make it a list instead, e.g. "Our proposed method was superior in three aspects: (1) superior in detecting causal variants without. . . ; (2) . . . ".

- P2. L39 “which is the computationally efficient” -> “which is a computationally efficient”
- P3. L77 “i_{th}” should be “i-th” (same goes for other ones).
- P7. L169 I suggest “adjusted $-\log_{10}(p)$” written as “$-\log_{10}(p_a)$” otherwise Eq (19) is too awkward to read.
- P8 L218 “as the R package” -> “as an R package”
