# Supplementary material for: RAINBOW: Haplotype-based genome-wide association study using a novel SNP-set method
Source: PLoS Comput Biol. 2020 Feb 14;16(2):e1007663. doi: 10.1371/journal.pcbi.1007663 (PMC7046296; doi:10.1371/journal.pcbi.1007663)
Supplement: Attachment — Submitted filename: Response_to_Reviewers.docx [file pcbi.1007663.s011.docx]

Graduate School of Agricultural and Life Sciences, The University of Tokyo
1-1-1 Yayoi, Bunkyo, Tokyo 113-8657
Tel.: +813-5841-5068

E-mail: aiwata@mail.ecc.u-tokyo.ac.jp

December 10, 2019

Dr. Mihaela Pertea

Software Editor, PLOS Computational Biology

Dear Dr. Pertea,

Re: Decision from PLOS Computational Biology (PCOMPBIOL-D-19-01767)

We would like to resubmit our revised manuscript entitled “RAINBOW: Haplotype-based genome-wide association study using a novel SNP-set method” (PCOMPBIOL-D-19-01767). According to the points raised by the Associate Editors and reviewers, we revised the manuscript as described in the following pages.

We thank you in advance for considering the revised manuscript for publication in the **PLOS Computational Biology** and look forward to hearing from you at your earliest convenience.

Yours Sincerely,

Hiroyoshi Iwata, Ph. D

Associate Professor,

Department of Agricultural and Environmental Biology,

Graduate School of Agriculture and Life Sciences,

The University of Tokyo

**Reply to the reviewers:**

**Reply to the Reviewer #1:**

**In general the materials and methods, Results and Discussion sections are well written; however, the abstract and introduction sections needs some improvements. Especially for describing better the scope and implications of the results of the proposed method.**

As the reviewer suggested, we should describe the scope and implications of the results of our proposed method. We added the scope of the results in the Introduction section (L51-58 in the revised manuscript).

**Here a few minor points.**

**Page 2, lines 6-9. sequencing data is the "3000 rice genomes project" [].**

**such public data, the conventional GWAS**

**Page 2, line 29. in false**

**as in the world collection of rice germplasm used in this**

**drawback: a weighting**

**Line 39. which is a computationally**

**method since it requires**

**Page 3, lines 47-49. Please rephrase.**

**Adding a diagram for explaining the proposed simulation scheme would help to understand better the results**

We modified the minor points that the reviewer suggested. Please refer to the P2, L7; P2, L10; P2, L29-32; P2, L39-40; P2, L47-50. We also created a diagram for explaining the proposed simulation scheme as S1 Fig. We changed the Supplemental Figure names because we create new diagram, S1 Fig, in the revised manuscript (L81-82).

**Reply to the Reviewer #2:**

**Their method to fit their model, which is rather ad-hoc, I suspect arise from the software restriction of EMMA/GEMMA that can only fit a single random effect model. The aspect of this fitting process that I question is *how good are the resulting estimates compared to say if the model parameters were estimated directly via REML*? Some existing software such as SAS and asreml are able to estimate variance components directly via REML even when there are multiple random effects. These are of course commercial software and likely authors would like to have RAINBOW without those restrictions but I think a comparison of variance component estimates that authors obtain to REML estimates are warranted.**

We compared our proposed method, RAINBOW, to the direct estimation approach via REML by using the lmm.aireml function in the R package gaston. To achieve this, we conducted a simple simulation study with 100 iterations using the marker genotype data of rice (Zhao et al., 2010; PLoS One 5(5): e10780). This data consists of 395 accessions with 1,311 SNPs. First, we prepared the additive relationship matrix and the epistatic (additive x additive) relationship matrix from marker genotype data. Then, we simulated phenotypic values by first sampling additive and epistatic genotypic values from the multivariate normal distribution whose covariance matrix is proportional to each relationship matrix and then adding residuals. We prepared 9 scenarios for heritability (0.1, 0.2, 0.3, 0.4, 0.5, 0.6, 0.7, 0.8, 0.9) and 4 scenarios for the weights between additive and epistatic effects (3:0, 2:1, 1:2, 0:3). We estimated variance components by RAINBOW and lmm.aireml and calculated log-likelihood. Then, we compared the full model with two kernels (additive and epistasis) to the null model with one kernel (additive) and calculated the deviances between two models.

The results showed that some deviances calculated by lmm.aireml (25%) were negative and failed to estimate variance components, especially when the heritability is too low/high (the weights between additive and epistatic effects did not affect the results very much). On the other hand, none of the deviances estimated by RAINBOW were negative. We think this is the advantage of our method, and we think this is partly because the search space of the weights is limited to the interval $[0, 1]$ in our method. Therefore, the convergence of our proposed fitting method is relatively warranted even when the heritability is too low/high.

We added the explanation of the advantage of our method described above to the “Estimation of variance components” subsection in the revised manuscript (L126-134).

**General comment:**

- $\boldsymbol{\rho}_{\boldsymbol{12}}$ **for Eqns (16) and (17) does not seem to be defined. I assumed to be Pearson’s correlation coefficient between** $\mathbf{X}_{\mathbf{1}}$ **and** $\mathbf{X}_{\mathbf{2}}$ **but could the authors clarify?**

We defined in L178, but just as “correlation”, so we redefined the parameter $\rho_{12}$ as “Pearson’s correlation coefficient”.

- **P4. L82 & L89 & L95.** $\boldsymbol{\sigma}_{\boldsymbol{c}}^{\boldsymbol{2}}$ **and** $\boldsymbol{\sigma}_{\boldsymbol{r}_{\boldsymbol{i}}}^{\boldsymbol{2}}$ **are not estimated by solving the mixed effect model Eq (1). It is important to distinguish between the *model* and the *fitting process* of the model. The fitting process estimates the variance parameters usually using REML, but not always the case as is the authors’ case described in “Estimation of variance components” section. The authors could just refer to “Estimation of variance components” section rather than referring that it is solved by model Eq (1).**

As the reviewer suggested, we should refer to “Estimation of variance components” section for the estimated variance components, and we fixed them. Please refer to L98, L105, and L112 in the revised manuscript.

- **Any reference to back up the statement “the score test is not necessarily the best method for testing the random effects in the mixed effects model” in L41?**

As written in L45-47, Lippert *et al*. (2014) reported that the LR test showed the greater power than the score test, then we referred to their paper for the L41-42.

**Citations:**

- **Please cite software used. E.g. cite R; and cite ggplot2 for creating the figures. Citations for R-packages are easily obtained in R by using say citation("ggplot2") for gglot2 R package.**

As the reviewer mentioned, we should cite some papers for some R packages. We added citations for the R, the R package ggplot2, Rcpp, and RcppEigen (L77-81).

- **Please cite relevant statistical papers. E.g. REML attributed to Patterson & Thompson (1971); testing of variance components at boundary -> the asymptotic distribution discussed in Self and Liang (1987) with further discussion in Stram and Lee (1994) (latter may be more relevant as it pertains directly to LR tests. Score test, ML and so on also missing citations.**

We added citations for the relevant statistical metods that the reviewer mentioned as follows.

For ML and REML: L115-116,

- Wilks SS. The Large-Sample Distribution of the Likelihood Ratio for Testing Composite Hypotheses. Ann Math Stat. 1938;9(1):60–62.
- Edgeworth FY. On the Probable Errors of Frequency-Constants (Contd.). J R Stat Soc. 1908;71(3):499–512.
- Patterson HD, Thompson R. Recovery of inter-block information when block sizes are unequal. Biometrika. 1971;58(3):545 –554.

For LR test: L42-43,

- Neyman J, Pearson ES. On the Use and Interpretation of Certain Test Criteria for Purposes of Statistical Inference. Biometrika. 1928;20A(1-2):175–240.
- Wilks SS. The Large-Sample Distribution of the Likelihood Ratio for Testing Composite Hypotheses. Ann Math Stat. 1938;9(1):60–62.

For testing of variance components at boundary: L146,

- Self SG, Liang KY. Asymptotic properties of maximum likelihood estimators and likelihood ratio tests under nonstandard conditions. J Am Stat Assoc. 1987;82(398):605–610.
- Stram DO, Lee JW. Variance Components Testing in the Longitudinal Mixed Effects Model. Biometrics. 1994;50(4):1171–1177.

For score test: L39,

- Rao CR. Large sample tests of statistical hypotheses concerning several parameters with applications to problems of estimation. Math Proc Cambridge Philos Soc. 1948;44(1):50–57.

For the direct estimation method via AIREML: L130-131,

- Gilmour AR, Thompson R, Cullis BR. Average Information REML: An Efficient Algorithm for Variance Parameter Estimation in Linear Mixed Models. Biometrics. 1995;51(4):1440–1450.
- Yang J, Lee SH, Goddard ME, Visscher PM. GCTA: A tool for genome-wide complex trait analysis. Am J Hum Genet. 2011;88(1):76–82.
- Covarrubias-Pazaran G. Genome-Assisted prediction of quantitative traits using the r package sommer. PLoS One. 2016;11(6):1–15.

For the convergence of two-step methods:

- Wang J, Do H, Woznica A, Kalousis A. Metric learning with multiple kernels. Adv Neural Inf Process Syst. 2011;1170–1178.

**Minor comments:**

**Abstract:**

- **“The results indicated that the proposed method was able to control false positives than the others.” -> control false positives *better* than other methods.**
- **“The proposed method was also excellent at detecting causal variants without relying on the linkage disequilibrium if causal variants were genotyped in the dataset. Moreover, the proposed method showed greater power than the other methods, i.e., it was able to detect causal variants that were not detected by the others, primarily when the causal variants were located very close to each other, and the directions of their effects were opposite. The proposed method, RAINBOW, is exceptionally superior in controlling false positives, detecting causal variants, and detecting nearby causal variants with opposite effects. By using the SNP-set approach as the proposed method, we expect that detecting not only rare variants but also genes with complex mechanisms, such as genes with multiple causal variants, can be realized.” -> Rather than repeating “the proposed method” so many times, I suggest you make it a list instead, e.g. “Our proposed method was superior in three aspects: (1) superior in detecting causal variants without. . . ; (2) ...”.**

We modified the Abstract as the reviewer suggested. Please check the highlighted sentences in the Abstract. (“Our poposed method was superior … By using the SNP-set approach as in this study,”)

- **P2. L39 “which is the computationally efficient” -> “which is a computationally efficient”**
- **P3. L77 “i_th” should be “i-th” (same goes for other ones).**
- **P7. L169 I suggest “adjusted** $\boldsymbol{-}\mathbf{log}_{\boldsymbol{10}} \boldsymbol{(p)}$**” written as “**$\boldsymbol{-}\mathbf{log}_{\boldsymbol{10}} \boldsymbol{(}\boldsymbol{p}_{\boldsymbol{a}}\boldsymbol{)}$**” otherwise Eq (19) is too awkward to read.**
- **P8 L218 “as the R package” -> “as an R package”**

We modified the minor points that the reviewer suggested. Please refer to the P2, L39; P3, L93, L102, L105, L107; P8, Eq (19), L204; P9, L246.

**Other minor changes in the revised manuscript**

- The package name of RAINBOW was changed into “RAINBOWR” (RAINBOW with R). This is because the original package name RAINBOW conflicted with the package [rainbow](https://cran.r-project.org/web/packages/rainbow/index.html) when we submitted our package to the [CRAN](https://cran.r-project.org/) (Comprehensive R Arichive Network). The R package RAINBOWR is now available at the CRAN. We dscribed this in the last sentence of the Abstract and in the “Availabiity of data and material” subsection (L248-251). We also add source codes of the R package RAINBOWR as a supporting information, S1 File, and we added this explanation to the “Availabiity of data and material” subsection (L252-253). With this change, we changed the file name for the Supplementary Note ( “S1 File” in the original manuscript) to “S1 Appendix”.
- In the Inroduction, we described the haplotype-based approaches in animal genomics, however, the desciription was not correct. We modified the description as the L64-66 in the revised manuscript.
- The author name was wrong (“Hiroyoshi Iata”) in the original manuscript, then we modified this to the correct one (“Hiroyoshi Iwata”).
